# Association of maternal education, neighborhood deprivation, and racial segregation with gestational age at birth by maternal race/ethnicity and United States Census region in the ECHO cohorts

**DOI:** 10.3389/fpubh.2023.1165089

**Published:** 2023-11-30

**Authors:** Anne L. Dunlop, Mohamad Burjak, Lorraine T. Dean, Akram N. Alshawabkeh, Lyndsay A. Avalos, Judy L. Aschner, Carrie V. Breton, Mia A. Charifson, Jose Cordero, Dana Dabelea, Viren D’Sa, Cristiane S. Duarte, Amy J. Elliott, Stephanie M. Eick, Assiamira Ferrara, Raina N. Fichorova, Jody M. Ganiban, James E. Gern, Monique M. Hedderson, Julie B. Herbstman, Alison E. Hipwell, Kathi C. Huddleston, Margaret Karagas, Catherine Karr, Jean M. Kerver, Daphne Koinis-Mitchell, Kristen Lyall, Juliette Madan, Carmen Marsit, Cindy T. McEvoy, John D. Meeker, Emily Oken, T. Michael O’Shea, Amy M. Padula, Sheela Sathyanarayana, Susan Schantz, Rebecca J. Schmidt, Jessica Snowden, Joseph B. Stanford, Scott Weiss, Robert O. Wright, Rosalind J. Wright, Xueying Zhang, Monica McGrath

**Affiliations:** ^1^Department of Gynecology and Obstetrics, Emory University School of Medicine, Atlanta, GA, United States; ^2^Department of Epidemiology, Johns Hopkins Bloomberg School of Public Health, Baltimore, MD, United States; ^3^Department of Civil and Environmental Engineering, College of Engineering, Northeastern University, Boston, MA, United States; ^4^Division of Research, Kaiser Permanente Northern California, Oakland, CA, United States; ^5^Albert Einstein College of Medicine, Bronx, NY, United States; ^6^Hackensack Meridian School of Medicine, Nutley, NJ, United States; ^7^Department of Population and Public Health Sciences, University of Southern California, Los Angeles, CA, United States; ^8^Division of Epidemiology, New York University Langone Health Grossman School of Medicine, New York, NY, United States; ^9^Department of Epidemiology and Biostatistics, University of Georgia College of Public Health, Athens, GA, United States; ^10^Lifecourse Epidemiology of Adiposity and Diabetes Center, University of Colorado Anschutz Medical Campus, Aurora, CO, United States; ^11^Department of Pediatrics, Rhode Island Hospital and The Warren Alpert Medical School of Brown University, Providence, RI, United States; ^12^Division of Child and Adolescent Psychiatry, Columbia University-New York State Psychiatric Institute, New York, NY, United States; ^13^Avera Research Institute, Department of Pediatrics, University of South Dakota School of Medicine, Sioux Falls, SD, United States; ^14^Gangarosa Department of Environmental Health, Rollins School of Public Health, Emory University, Atlanta, GA, United States; ^15^Department of Obstetrics, Gynecology and Reproductive Biology, Brigham and Women’s Hospital, Harvard Medical School, Boston, MA, United States; ^16^Department of Psychological and Brain Sciences, George Washington University, Washington, DC, United States; ^17^Department of Pediatrics, School of Medicine and Public Health, University of Wisconsin-Madison, Madison, WI, United States; ^18^Department of Environmental Health Sciences, Columbia University Mailman School of Public Health, New York, NY, United States; ^19^Department of Psychiatry, University of Pittsburgh, Pittsburgh, PA, United States; ^20^College of Health and Human Services, George Mason University, Fairfax, VA, United States; ^21^Department of Epidemiology, Geisel School of Medicine at Dartmouth, Hanover, NH, United States; ^22^Departments of Pediatrics and Environmental and Occupational Health Sciences, University of Washington, Seattle, WA, United States; ^23^Departments of Epidemiology and Biostatistics and Pediatrics and Human Development, College of Human Medicine, Michigan State University, East Lansing, MI, United States; ^24^AJ Drexel Autism Institute, Drexel University, Philadelphia, PA, United States; ^25^Department of Epidemiology, Pediatrics and Psychiatry, Geisel School of Medicine at Dartmouth, Hanover, NH, United States; ^26^Department of Pediatrics, Oregon Health and Science University, Portland, OR, United States; ^27^Department of Environmental Health Sciences, University of Michigan School of Public Health, Ann Arbor, MI, United States; ^28^Division of Chronic Disease Research Across the Lifecourse, Department of Population Medicine, Harvard Medical School and Harvard Pilgrim Health Care Institute, Boston, MA, United States; ^29^Department of Pediatrics, University of North Carolina at Chapel Hill, Chapel Hill, NC, United States; ^30^Department of Obstetrics, Gynecology and Reproductive Sciences, University of California, San Francisco, San Francisco, CA, United States; ^31^Beckman Institute for Advanced Science and Technology, University of Illinois at Urbana-Champaign, Urbana, IL, United States; ^32^Department of Public Health Sciences, MIND Institute, University of California, Davis, Davis, CA, United States; ^33^Departments of Pediatrics and Biostatistics, University of Arkansas for Medical Sciences, Little Rock, AR, United States; ^34^Department of Family and Preventive Medicine, University of Utah School of Medicine, Salt Lake City, UT, United States; ^35^Department of Medicine, Harvard School of Medicine, Boston, MA, United States; ^36^Department of Pediatrics, The Kravis Children’s Hospital, Icahn School of Medicine at Mount Sinai, New York, NY, United States; ^37^Institute for Exposomic Research, Icahn School of Medicine at Mount Sinai, New York, NY, United States

**Keywords:** gestational age, premature birth, residential segregation, socioeconomic status, racial and ethnic health disparities, ECHO program

## Abstract

**Background:**

In the United States, disparities in gestational age at birth by maternal race, ethnicity, and geography are theorized to be related, in part, to differences in individual- and neighborhood-level socioeconomic status (SES). Yet, few studies have examined their combined effects or whether associations vary by maternal race and ethnicity and United States Census region.

**Methods:**

We assembled data from 34 cohorts in the Environmental influences on Child Health Outcomes (ECHO) program representing 10,304 participants who delivered a liveborn, singleton infant from 2000 through 2019. We investigated the combined associations of maternal education level, neighborhood deprivation index (NDI), and Index of Concentration at the Extremes for racial residential segregation (ICE_Race_) on gestational weeks at birth using linear regression and on gestational age at birth categories (preterm, early term, post–late term relative to full term) using multinomial logistic regression.

**Results:**

After adjustment for NDI and ICE_Race_, gestational weeks at birth was significantly lower among those with a high school diploma or less (−0.31 weeks, 95% CI: −0.44, −0.18), and some college (−0.30 weeks, 95% CI: −0.42, −0.18) relative to a master’s degree or higher. Those with a high school diploma or less also had an increased odds of preterm (aOR 1.59, 95% CI: 1.20, 2.10) and early term birth (aOR 1.26, 95% CI: 1.05, 1.51). In adjusted models, NDI quartile and ICE_Race_ quartile were not associated with gestational weeks at birth. However, higher NDI quartile (most deprived) associated with an increased odds of early term and late term birth, and lower ICE_Race_ quartile (least racially privileged) associated with a decreased odds of late or post-term birth. When stratifying by region, gestational weeks at birth was lower among those with a high school education or less and some college only among those living in the Northeast or Midwest. When stratifying by race and ethnicity, gestational weeks at birth was lower among those with a high school education or less only for the non-Hispanic White category.

**Conclusion:**

In this study, maternal education was consistently associated with shorter duration of pregnancy and increased odds of preterm birth, including in models adjusted for NDI and ICE_Race_.

## 1 Introduction

Gestational age at birth is a critical determinant of child health and development (1). It is well-established that preterm birth (before 37 completed gestational weeks) markedly increases the risk for infant and early childhood mortality and serious morbidity (2). More recently, it has become clear that each additional week of gestation before and after the full term period (39 through 40 completed weeks) increases the risk for infant morbidity and mortality (3). Those born late preterm (34 through 36 completed weeks) and early term (37 through 38 completed weeks) experience an increased risk for infant mortality and morbidity (4–7) relative to those born full term. Moreover, both late term (41 through 41\u00B0completed weeks) and post-term (42 completed weeks and beyond) birth increase the risk for stillbirth and perinatal death and confer risk for macrosomia and related birth complications, meconium aspiration, and neonatal seizures (3, 8).

Gestational age at birth is also an important determinant of cognitive and educational outcomes. Systematic reviews demonstrate that, relative to their full term peers, children born preterm are at increased risk for academic difficulties in reading and math (9) and those born early term are at increased risk for adverse cognitive and educational outcomes (10). A study of more than 1 million children in the United States (U.S.) found that those born late term had a 3.1% reduced risk of poor cognitive outcomes and a 2.8% higher probability of being labeled as “gifted” compared with their full term peers but were 2.1% more likely to have a physical impairment (11). A large study of more than 400,000 school children in the United Kingdom (UK) concluded that gestational age at birth accounted for 10% of the adjusted population-attributable fraction of special education needs (12).

Within the United States, there are stark disparities in gestational age at birth, including by maternal race and ethnicity (7, 13). From 2015 to 2017, the highest rate of preterm birth in the United States was among those who identified as non-Hispanic Black (13.6%), followed by American Indian/Alaska Native (11.3%), Hispanic (9.4%), non-Hispanic White (9.0%), and Asian/Pacific Islander (8.7%) (14). A recent study in northern California, however, found that Asian participants had an elevated rate of preterm birth relative to non-Hispanic White participants (15), suggesting that geographic variations in racial and ethnic disparities in gestational age at birth may exist. Additionally, rates of preterm birth vary across states, with the lowest rates in northeastern and northwestern states and the highest rates in southeastern states (16, 17). According to the Center for American Progress (18), there is wide variation across states in a range of policies that affect access to health care and human services, which shape both utilization of health care and the prevalence of health behaviors and conditions linked to birth outcomes.

Racial, ethnic, and geographic disparities in gestational age at birth are theorized to be related, in part, to differences in individual-level socioeconomic status (SES) (19). Those of lower SES have a higher burden of adverse health outcomes across the life course (20, 21). In fact, there is a consistent social gradient in the risk of preterm birth across several measures of individual-level SES, including maternal education and income levels, marital and employment status, and health insurance type (19). Differences in these individual-level SES measures do not, however, entirely account for observed disparities in preterm birth (22) and have not been well studied for other gestational age categories. Of note, traditional measures of SES (such as education and income) may not fully capture an individual’s SES, which is likely influenced by immigration and acculturation status and English proficiency, among other factors (23).

Differences in neighborhood-level SES are increasingly recognized as contributors to disparities in health outcomes (24, 25), including gestational age at birth (26–35). Neighborhood-level SES is a construct that encompasses resource allocation, social marginalization, and power exchange, which are reflected in measures of deprivation and segregation (36). The legacy of United States segregation (both de jure and *de facto*) via historical and contemporary systemic racism (37, 38) continues to impact racial and ethnic minorities to this day, especially for Black Americans who have fewer socioeconomic opportunities, are differentially exposed to lifelong financial stress, and are more likely to reside in neighborhoods with concentrated poverty, infrastructure decay, resource divestment, and environmental hazards (39, 40). It is notable that gains in individual-level SES do not protect Black individuals from residential racial segregation (41). Neighborhood-level measures of SES have been conceptually linked to adverse birth outcomes via pathways mediated by individual-level health behaviors, psychosocial factors, social support, stress, and access to quality health care, food, and recreational facilities (42).

Understanding the factors that drive socioeconomically patterned inequalities in gestational age at birth requires socioeconomic data from various ecological levels (36, 43). However, few studies have investigated the *combined* effect of individual- and neighborhood-level measures of SES on gestational age at birth (44) or whether any observed associations vary by maternal race and ethnicity and geography of residence. To fill this gap, we capitalized on data from the large, diverse nationwide sample enrolled in the Environmental influences on Child Health Outcomes (ECHO) program to investigate these relationships. Specifically, using ECHO data, we sought to examine associations of maternal education, as an individual-level measure of SES, in conjunction with neighborhood-level deprivation and racial segregation, with gestational age at birth overall and stratified by maternal race and ethnicity and United States Census region. A range of maternal health conditions and health behaviors may be on the causal pathway between SES exposures and gestational age at birth; therefore, we considered whether variables that capture specific maternal health conditions and health behaviors influence any observed associations.

## 2 Methods

### 2.1 Design

This study combined data from multiple United States pregnancy and pediatric cohorts in the ECHO program. In 2016, the National Institutes of Health (NIH) launched the ECHO program to investigate the influence of early life environmental exposures on child health and development. ECHO supports pediatric cohorts throughout the United States in the sharing of extant data and the collection of new data under a common protocol. This nationwide consortium is able to leverage demographic and geographic heterogeneity and a large sample size to answer important research questions about child health and development (45, 46). The ECHO study protocol was approved by the local and/or central ECHO Institutional Review Board. Written informed consent was obtained from parents/caregivers (along with child assent as age-appropriate).

### 2.2 Study population

**Figure 1:**
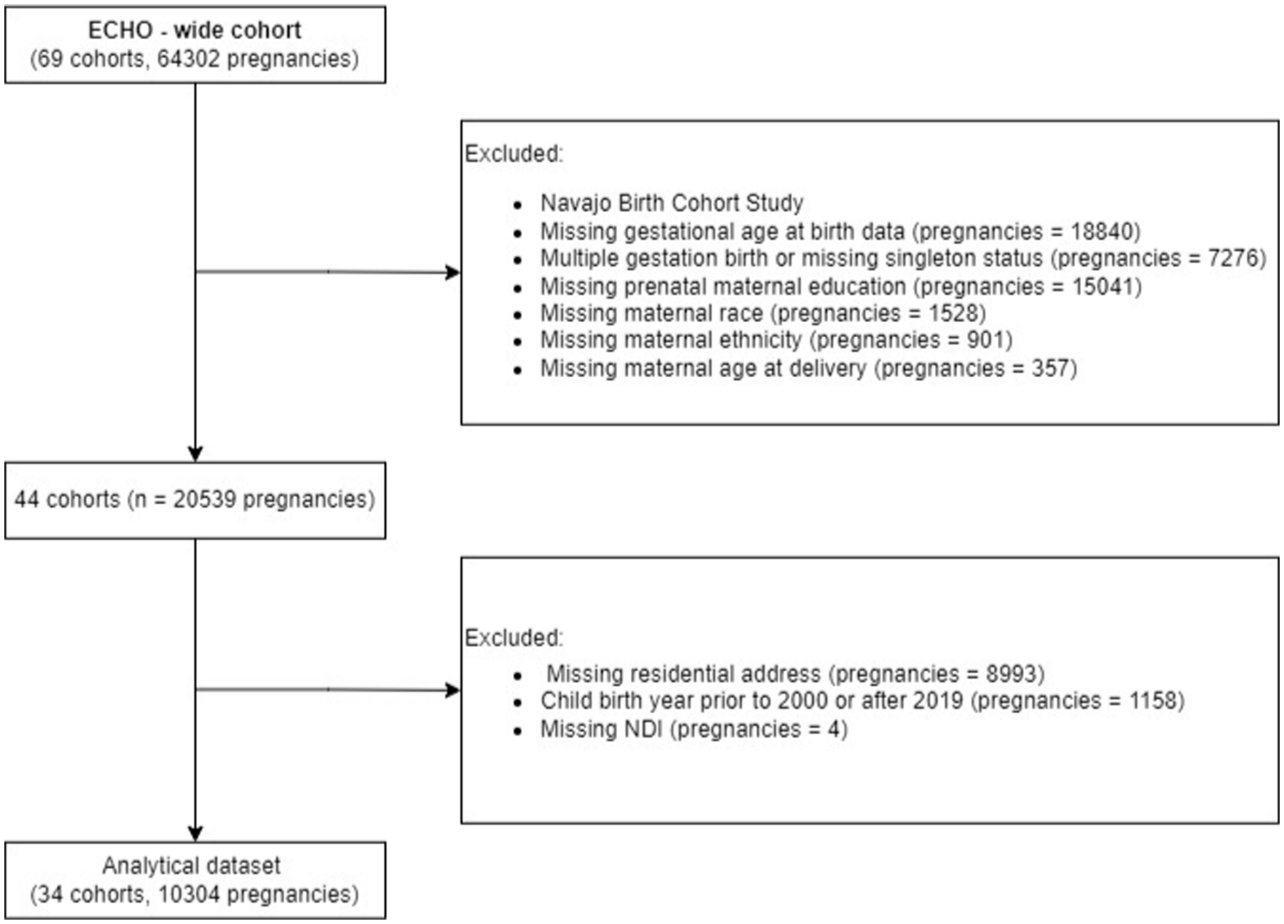
Derivation of the study sample from ECHO cohort data.

The primary study population consisted of 10,304 participants from 34 ECHO cohorts who delivered a liveborn, singleton infant from 2000 through 2019 and who had the following outcome and exposure information available for analysis: gestational age at birth, maternal prenatal level of education, maternal race and ethnicity, and at least one geocoded residential address during pregnancy (Figure 1). We restricted inclusion to those cohorts with available data on at least 20 observations (*n* = 8 cohorts were dropped). The secondary study population excluded three cohorts (*n* = 547) that enrolled only preterm births (< 37 weeks) and included 9,757 participants from 31 ECHO cohorts. This secondary study population was utilized to investigate the association between the exposures and gestational age outcome categories.

### 2.3 Outcome measure–gestational age at birth

Gestational age at birth in completed weeks was obtained through abstraction of maternal or child medical records or through parent report. For medical record abstraction, an accepted hierarchy was employed to ascertain the most accurate measure for estimating the due date: dating based on embryo placement following *in vitro* fertilization or on artificial insemination (0%), obstetrical estimate from first trimester ultrasound (2%), obstetrical estimate from ultrasound taken in the second trimester with fetal biparietal diameter dating within 2 weeks of sure last menstrual period (LMP) (0%), ultrasound taken in the second trimester with unsure or no LMP date (0%), report from obstetrical medical record reporting “consensus” estimated date of delivery with no ultrasound documented during first and second trimester (8%), obstetrical estimate from LMP only (8%), neonatal estimate of gestational age at birth obtained from child medical records (39%), estimate from cohort research encounter (3%), report by mother (27%), and cohort-provided estimated date of delivery without further description (12%) (47, 48).

Gestational age at birth was assessed as a *continuous measure* (gestational weeks) and a *categorical measure* (preterm, 22–0/7 through 36–6/7 weeks; early-term, 37–0/7 through 38–6/7 weeks; full-term, 39–0/7 through 40–6/7 weeks [reference category]; and late- and post-term, 41–0/7 to 43–0/7 weeks). These categorizations were based on the definitions of the American College of Obstetricians and Gynecologists Committee Opinion (49).

### 2.4 Individual-level SES measure–maternal education

Existing research among United States populations suggests that education is the dimension of SES that most strongly and consistently predicts maternal and child health outcomes (50, 51). Maternal self-reported highest level of education during the prenatal period served as the principal individual-level measure of SES and was categorized as: (1) up to a high school diploma or equivalent; (2) some college (no degree), an associate’s degree, or trade school; (3) a bachelor’s degree; and (4) a master’s degree and above (reference category).

### 2.5 Neighborhood-level SES measures–deprivation and racial segregation

Participant self-reported addresses were geocoded in ArcGIS Pro Streetmap Premium Geocoder. Streetmap Premium includes the underlying locational databases (streets, parcels, rooftops, administrative boundaries) that addresses match to and the algorithms and code that attempt to select the most accurate latitude/longitude location for each address (52). To assess the reliability of addresses, a geocoding quality variable was constructed based on ECHO’s ability to match addresses to a point address with values ranging from 1 to 9. Our inclusion criteria required a level of 6 or lower for geocoding quality to be included in the analysis. In our study population, over 99% of addresses had a high-quality match of level 4 or lower (point or specific street address). We assigned a census tract identifier to each participant address using the appropriate census tract boundary file. When more than one address was available for a given participant during the prenatal period, we used the first reported address during the pregnancy period; more than half of our study population had only one address available during the relevant time period. We identified 5,190 census tracts for the study population representing 45 states. Federal Information Processing Standards (FIPS) codes were then identified. Neighborhood-level measures were obtained by linking the FIPS codes to the 2000 decennial censuses and the American Community Survey (ACS) 5-Year Estimates for 2010–2019 depending on the child’s year of birth. Specifically, the neighborhood-level measures were calculated (as described below) for all census years used in this study, meaning for the 2000 decennial census and all ACS 5-year surveys from 2010 to 2019. Study participants were then matched with a neighborhood-level measure value based on the census tract they were residing in during pregnancy. If the pregnancy was prior to 2010, the participant was assigned a value based on the 2000 decennial census using their year 2000 census tract. For participants whose pregnancy was in 2010 and beyond, they were assigned a value based on the corresponding ACS year using their year 2010 census tract.

Using published formulae, we calculated two established indices to serve as the principal neighborhood-level measures of SES: the Neighborhood Deprivation Index (NDI) (53) and the Index of Concentrations at the Extremes for racial residential segregation (ICE_Race_) (54, 55). We chose NDI because it is a multi-dimensional, composite index that summarizes neighborhood deprivation (53), and previous research has associated this measure with preterm birth (26), including among non-Hispanic Black and White women in eight geographic areas in the United States (28). We chose to focus on racial residential segregation, as measured by ICE_Race_, because growing literature suggests that racial segregation is an important driver of health disparities, including preterm birth and infant mortality (40). We calculated these indices at the level of census tract based on literature showing that census tract-level analyzes resulted in maximal geocoding linkage (i.e., the highest proportion of records geocoded and linked to census-defined geography) and that measures of economic deprivation at the census tract-level were most sensitive to expected socioeconomic gradients in health among non-Hispanic Black, non-Hispanic White, and Hispanic men and women (56, 57).

NDI is a summary measure created through principal components analysis using the following eight census tract measures (as percentages): males in management and professional occupations, crowded housing, households in poverty, female-headed households with dependents, households on public assistance, households earning < $30,000 per year, adults with less than a high school education, and unemployed individuals (53). The census tracts were then matched to their corresponding NDI value based on the year of the birth of the child. Higher NDI values indicate a higher level of socioeconomic deprivation in the census tract. In our analysis, NDI values were categorized into quartiles based on the distribution of our analytical sample, with the fourth quartile (highest NDI) representing the highest level of socioeconomic deprivation in the census tract, consistent with the published literature (52) and for ease of interpretability.

ICE_Race_ was utilized to quantify the extent of racial segregation within the census tract and was calculated using the established formula (54, 55):


$$ICE_{i}=\left(A_{i}-P_{i}\right)/T_{i}$$

Where for a given census tract i:

A_i_ = the number of persons in the most racially privileged category in the census tract.

P_i_ = the number of persons in the least racially privileged category in the census tract.

T_i_ = the total count of persons (of all races and ethnicities) in the census tract.

For this analysis, the most racially privileged category was defined as non-Hispanic White persons and the least racially privileged category was defined as non-Hispanic Black persons. ICE_Race_ ranges from −1 to 1; a value of 1 signifies that 100% of the population of the census tract is concentrated in the most racially privileged group, whereas a value of −1 signifies that 100% of the population of the census tract is concentrated in the least racially privileged group. The census tracts were matched to their corresponding ICE_Race_ value and categorized into quartiles, with the first quartile (lowest proportion of non-Hispanic White persons) representing those least racially privileged.

### 2.6 Stratifying variable–race and ethnicity

Maternal race and ethnicity were ascertained *via* self-report and then categorized as non-Hispanic White, non-Hispanic Black, non-Hispanic Other Race, and Hispanic. Non-Hispanic Other Race included non-Hispanic persons who identified their race as American Indian or Alaska Native, Native Hawaiian or other Pacific Islander, Asian, multiple races, or other race; these were collapsed into a single category due to small sample size.

### 2.7 Stratifying variable–United States Census region

**Figure 2:**
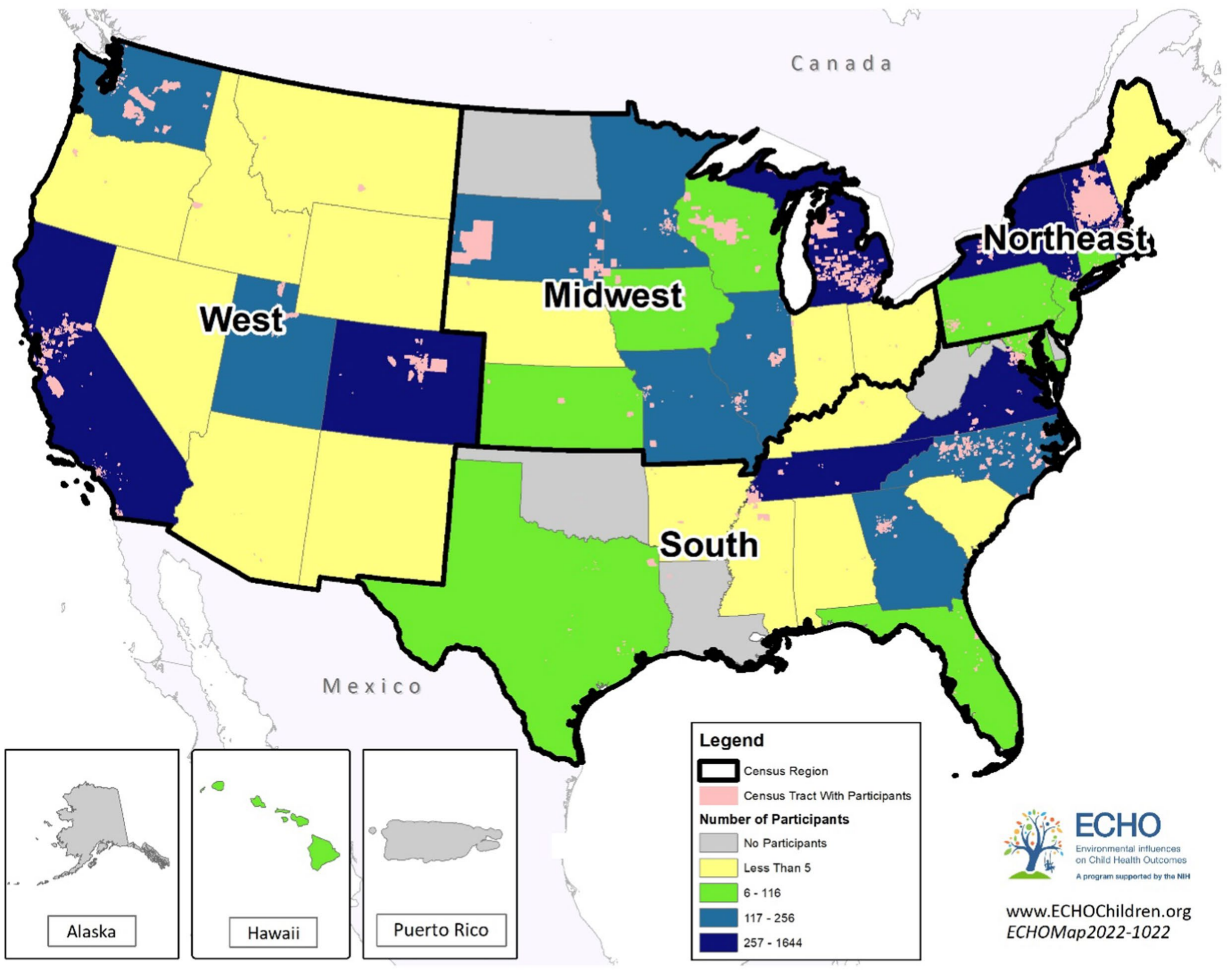
Prenatal residential census tracts and regions for the ECHO study sample.

Geographic region was assigned according to United States Census regions (Figure 2) categorized as Midwest, Northeast, South, or West, considering the state in which the mother resided during the pregnancy.

### 2.8 Covariates

Covariates for inclusion in statistical modeling were selected based on their association with SES and gestational age at birth in the literature (19, 56, 58) and their availability within the ECHO cohorts; the covariates that were included in statistical modeling are described in the next sections.

Maternal age in years (continuous), child sex (male, female), and parity (categorized as nulliparous, 1–2 births, and 3+ births prior to the index pregnancy) were based on medical record abstraction or maternal report. Prenatal marital status was based on maternal report and was categorized as married or living with a partner; widowed, separated, or divorced; or single, never married, and partnered not living together.

Pre-pregnancy body mass index (BMI) was defined using measured or self-reported height and weight between 12 months prior to conception through the first trimester and was categorized as underweight, healthy weight, overweight or obese according to accepted definitions (59). Gestational diabetes was defined as new-onset diabetes during pregnancy based on self-report or as indicated in medical records. The presence of preeclampsia or eclampsia, gestational hypertension, or gestational diabetes during the index pregnancy was ascertained *via* self-report or medical record abstraction with documentation of high blood pressure or anti-hypertensive medication use during pregnancy.

Prenatal substance use was ascertained through self-report or medical record abstraction for substance use during pregnancy. Binary variables (yes/no) for tobacco, alcohol, and marijuana use during pregnancy were created. Nicotine exposure was defined as cigarette smoking, use of Electronic Nicotine Delivery Devices/ENDS (e-cigarettes, vapes, vape pens, etc.), and other forms of tobacco (chewing tobacco/snuff, nicotine patch, nicotine gum/lozenges, cigar, pipe, hookah, Bidi/Beedi). Alcohol use included the consumption of beer, wine, mixed drinks, spirits, shot liquor, or any other type of alcohol.

### 2.9 Statistical analyzes

We described maternal sociodemographic and health characteristics overall and by maternal education categories, tabulating means and standard deviations (SDs) for continuous variables and the number of observations, missingness, and the percentage of total observations for categorical variables (Table 1). Additionally, we described maternal education level, NDI quartile, and ICE_Race_ according to maternal race and ethnicity categories and United States Census regions (Tables 2, 3). To evaluate the associations between maternal education, neighborhood deprivation, and racial segregation with completed gestational weeks in the primary study population, we performed a linear mixed effects model with random intercepts for cohort membership to account for within-cohort correlation (60). We employed multinomial logistical regression to estimate odds ratios (ORs) and 95% confidence intervals (Cis), considering categories of completed gestational weeks (preterm, early-term, late- and post-term) as the outcome in relation to full-term birth (reference category) after excluding the three cohorts that enrolled only preterm children. We calculated intraclass coefficients (ICCs) to assess cohort effects; all ICCs were < 0.02 indicating that no significant variation in the outcome was due to between-cohort differences in the secondary study population.

**Table 1:** Characteristics of ECHO cohort participants in the study sample overall and according to prenatal maternal level of education.

	Up to high school degree/GED	Some college no degree; associate degree; trade school	Bachelor’s degree (BA, BS)	Master’s degree, professional, or doctorate degree	Overall
	(*n* = 2559)	(*n* = 2329)	(*n* = 2817)	(*n* = 2599)	(*n* = 10304)
Gestational age (GA)					
Mean (SD)	37.6 (3.96)	37.7 (3.97)	38.5 (2.96)	38.7 (2.56)	38.2 (3.43)
Median [Min, Max]	39.0 [22.0, 43.0]	39.0 [22.0, 43.0]	39.0 [22.0, 42.0]	39.0 [23.0, 42.0]	39.0 [22.0, 43.0]
Estimated GA category					
Extremely preterm (22–27 weeks)	174 (6.8%)	154 (6.6%)	90 (3.2%)	53 (2.0%)	471 (4.6%)
Very preterm (28–31 weeks)	58 (2.3%)	55 (2.4%)	29 (1.0%)	18 (0.7%)	160 (1.6%)
Moderate preterm (32–33 weeks)	29 (1.1%)	25 (1.1%)	18 (0.6%)	17 (0.7%)	89 (0.9%)
Late preterm (34–36 weeks)	170 (6.6%)	145 (6.2%)	130 (4.6%)	129 (5.0%)	574 (5.6%)
Early term (37–38 weeks)	606 (23.7%)	475 (20.4%)	558 (19.8%)	520 (20.0%)	2,159 (21.0%)
Full term (39–40 weeks)	1,272 (49.7%)	1,243 (53.4%)	1,603 (56.9%)	1,488 (57.3%)	5,606 (54.4%)
Late term (41 weeks)	223 (8.7%)	214 (9.2%)	355 (12.6%)	327 (12.6%)	1,119 (10.9%)
Post term (42–43 weeks)	27 (1.1%)	18 (0.8%)	34 (1.2%)	47 (1.8%)	126 (1.2%)
Neighborhood deprivation index (NDI)					
Mean (SD)	0.79 (1.17)	0.24 (1.15)	−0.47 (0.82)	−0.69 (0.70)	−0.053 (1.14)
Median [Min, Max]	0.76 [−1.73, 5.35]	−0.075 [−1.75, 5.27]	−0.69 [−1.75, 4.22]	−0.86 [−1.80, 3.30]	−0.40 [−1.80, 5.35]
NDI category					
1st Quartile	149 (5.8%)	325 (14.0%)	929 (33.0%)	1,173 (45.1%)	2,576 (25.0%)
2nd Quartile	336 (13.1%)	547 (23.5%)	919 (32.6%)	774 (29.8%)	2,576 (25.0%)
3rd Quartile	676 (26.4%)	716 (30.7%)	679 (24.1%)	505 (19.4%)	2,576 (25.0%)
4th Quartile	1,398 (54.6%)	741 (31.8%)	290 (10.3%)	147 (5.7%)	2,576 (25.0%)
Index of concentrations at the extremes for racial segregation (ICE_Race_)					
Mean (SD)	0.086 (0.60)	0.33 (0.57)	0.58 (0.44)	0.62 (0.36)	0.41 (0.54)
Median [Min, Max]	0.10 [−1.00, 0.99]	0.41 [−1.00, 1.00]	0.73 [−0.99, 1.00]	0.72 [−0.98, 1.00]	0.55 [−1.00, 1.00]
ICE_Race_ Category					
1st Quartile	1,238 (48.4%)	759 (32.6%)	378 (13.4%)	201 (7.7%)	2,576 (25.0%)
2nd Quartile	640 (25.0%)	560 (24.0%)	686 (24.4%)	690 (26.5%)	2,576 (25.0%)
3rd Quartile	367 (14.3%)	452 (19.4%)	768 (27.3%)	989 (38.1%)	2,576 (25.0%)
4th Quartile	314 (12.3%)	558 (24.0%)	985 (35.0%)	719 (27.7%)	2,576 (25.0%)
Urbanicity					
Rural	251 (9.8%)	367 (15.8%)	664 (23.6%)	472 (18.2%)	1754 (17.0%)
Urban	2,308 (90.2%)	1962 (84.2%)	2,153 (76.4%)	2,127 (81.8%)	8,550 (83.0%)
United States Census Region					
Midwest	436 (17.0%)	509 (21.9%)	351 (12.5%)	343 (13.2%)	1,639 (15.9%)
Northeast	1,010 (39.5%)	886 (38.0%)	1,243 (44.1%)	1,103 (42.4%)	4,242 (41.2%)
South	621 (24.3%)	235 (10.1%)	358 (12.7%)	326 (12.5%)	1,540 (14.9%)
West	492 (19.2%)	699 (30.0%)	865 (30.7%)	827 (31.8%)	2,883 (28.0%)
Maternal race					
American Indian, Alaskan Native	32 (1.3%)	24 (1.0%)	<10	<5	69 (0.7%)
Asian	24 (0.9%)	72 (3.1%)	214 (7.6%)	261 (10.0%)	571 (5.5%)
Black	981 (38.3%)	579 (24.9%)	245 (8.7%)	113 (4.3%)	1918 (18.6%)
Multiple Races	121 (4.7%)	138 (5.9%)	91 (3.2%)	75 (2.9%)	425 (4.1%)
Native Hawaiian, Pacific Islander	16 (0.6%)	12 (0.5%)	<6	<5	37 (0.4%)
Other race	378 (14.8%)	184 (7.9%)	73 (2.6%)	48 (1.8%)	683 (6.6%)
White	1,007 (39.4%)	1,320 (56.7%)	2,180 (77.4%)	2094 (80.6%)	6,601 (64.1%)
Maternal ethnicity					
Hispanic	884 (34.5%)	585 (25.1%)	260 (9.2%)	165 (6.3%)	1894 (18.4%)
Non-Hispanic	1,675 (65.5%)	1744 (74.9%)	2,557 (90.8%)	2,434 (93.7%)	8,410 (81.6%)
Race/Ethnicity					
Hispanic	884 (34.5%)	585 (25.1%)	260 (9.2%)	165 (6.3%)	1894 (18.4%)
Non-Hispanic black	921 (36.0%)	525 (22.5%)	223 (7.9%)	110 (4.2%)	1779 (17.3%)
Non-Hispanic other^1^	162 (6.3%)	203 (8.7%)	307 (10.9%)	342 (13.1%)	1,014 (9.9%)
Non-Hispanic white	592 (23.1%)	1,016 (43.6%)	2027 (72.0%)	1982 (76.3%)	5,617 (54.5%)
Maternal age at delivery, years					
Mean (SD)	26.7 (5.95)	29.1 (5.33)	32.1 (4.46)	33.8 (3.80)	30.5 (5.64)
Median [Min, Max]	26.0 [14.0, 46.0]	29.0 [18.0, 46.0]	32.0 [20.0, 50.0]	34.0 [22.0, 52.0]	31.0 [14.0, 52.0]
Prenatal nicotine use^2^					
Missing	320 (12.5%)	316 (13.6%)	262 (9.3%)	294 (11.3%)	1,192 (11.6%)
No	1939 (75.8%)	1838 (78.9%)	2,505 (88.9%)	2,281 (87.8%)	8,563 (83.1%)
Yes	300 (11.7%)	175 (7.5%)	50 (1.8%)	24 (0.9%)	549 (5.3%)
Prenatal alcohol use^3^					
Missing	508 (19.9%)	406 (17.4%)	346 (12.3%)	343 (13.2%)	1,603 (15.6%)
No	1835 (71.7%)	1,581 (67.9%)	1913 (67.9%)	1718 (66.1%)	7,047 (68.4%)
Yes	216 (8.4%)	342 (14.7%)	558 (19.8%)	538 (20.7%)	1,654 (16.1%)
Prenatal marijuana use					
Missing	999 (39.0%)	893 (38.3%)	904 (32.1%)	984 (37.9%)	3,780 (36.7%)
No	1,383 (54.0%)	1,301 (55.9%)	1850 (65.7%)	1,585 (61.0%)	6,119 (59.4%)
Yes	177 (6.9%)	135 (5.8%)	63 (2.2%)	30 (1.2%)	405 (3.9%)
Pre-pregnancy maternal BMI, kg/m^2^					
Underweight (<18.5)	84 (3.3%)	53 (2.3%)	66 (2.3%)	61 (2.3%)	264 (2.6%)
Healthy Weight (18.5–24.9)	730 (28.5%)	748 (32.1%)	1,364 (48.4%)	1,539 (59.2%)	4,381 (42.5%)
Overweight (25.0–29.9)	516 (20.2%)	533 (22.9%)	642 (22.8%)	525 (20.2%)	2,216 (21.5%)
Obese (≥ 30)	767 (30.0%)	705 (30.3%)	535 (19.0%)	283 (10.9%)	2,290 (22.2%)
Missing	462 (18.1%)	290 (12.5%)	210 (7.5%)	191 (7.3%)	1,153 (11.2%)
Parity					
Nulliparous	704 (27.5%)	655 (28.1%)	1,121 (39.8%)	1,163 (44.7%)	3,643 (35.4%)
1–2	1,030 (40.3%)	1,023 (43.9%)	1,179 (41.9%)	1,064 (40.9%)	4,296 (41.7%)
>2	284 (11.1%)	251 (10.8%)	179 (6.4%)	72 (2.8%)	786 (7.6%)
Missing	541 (21.1%)	400 (17.2%)	338 (12.0%)	300 (11.5%)	1,579 (15.3%)
Child sex					
Female	1,413 (50.2%)	1,272 (48.9%)	1,139 (48.9%)	1,234 (48.2%)	5,058 (49.1%)
Male	1,404 (49.8%)	1,325 (51.0%)	1,188 (51.0%)	1,321 (51.6%)	5,238 (50.8%)
Missing	0 (0%)	2 (0.1%)	2 (0.1%)	4 (0.2%)	8 (0.1%)
Gestational hypertension					
Missing	1,177 (46.0%)	885 (38.0%)	870 (30.9%)	819 (31.5%)	3,751 (36.4%)
No	1,282 (50.1%)	1,330 (57.1%)	1803 (64.0%)	1,673 (64.4%)	6,088 (59.1%)
Yes	100 (3.9%)	114 (4.9%)	144 (5.1%)	107 (4.1%)	465 (4.5%)
Gestational diabetes in index pregnancy					
Missing	355 (13.9%)	262 (11.2%)	194 (6.9%)	185 (7.1%)	996 (9.7%)
No	1996 (78.0%)	1890 (81.2%)	2,413 (85.7%)	2,238 (86.1%)	8,537 (82.9%)
Yes	208 (8.1%)	177 (7.6%)	210 (7.5%)	176 (6.8%)	771 (7.5%)
(Pre) Eclampsia in index pregnancy					
Missing	559 (21.8%)	540 (23.2%)	481 (17.1%)	513 (19.7%)	2093 (20.3%)
No	1862 (72.8%)	1,655 (71.1%)	2,208 (78.4%)	1982 (76.3%)	7,707 (74.8%)
Yes	138 (5.4%)	134 (5.8%)	128 (4.5%)	104 (4.0%)	504 (4.9%)
Marital status					
Married or cohabiting	1,349 (52.7%)	1,688 (72.5%)	2,567 (91.1%)	2,485 (95.6%)	8,089 (78.5%)
Widowed; Separated; Divorced	171 (6.7%)	145 (6.2%)	71 (2.5%)	27 (1.0%)	414 (4.0%)
Single, never married; Partnered, not living together	896 (35.0%)	416 (17.9%)	153 (5.4%)	54 (2.1%)	1,519 (14.7%)
Missing	143 (5.6%)	80 (3.4%)	26 (0.9%)	33 (1.3%)	282 (2.7%)

^1^Non-Hispanic Other Race included non-Hispanic persons who identified their race as American Indian or Alaska Native, Native Hawaiian or other Pacific Islander, Asian, Multiple Races, or Other Race. ^2^Nicotine use included cigarette smoking, use of electronic nicotine delivery devices/ENDS (e-cigarettes, vapes, vape pens, etc.), and other forms of tobacco (including chewing tobacco/snuff, nicotine patch, nicotine gum/lozenges, cigar, pipe, hookah, Bidi/Beedi). ^3^Alcohol use included the consumption of beer, wine, mixed drink, spirits, shot liquor, or any other type of alcohol. BA, Bachelor of Arts; BS, Bachelor of Science; GED, General Educational Development.

**Table 2:** Maternal level of education, neighborhood deprivation index quartile, and index of concentrations of the extremes for race quartile in the ECHO cohort by maternal race and ethnicity.

	Non-Hispanic white	Non-Hispanic black	Non-Hispanic other	Hispanic	Overall
	(*n* = 5,617)	(*n* = 1779)	(*n* = 1,014)	(*n* = 1894)	(*n* = 10,304)
Neighborhood deprivation index, *n* (%)					
4th Quartile (most deprived)	328 (5.8%)	1,106 (62.2%)	191 (18.8%)	951 (50.2%)	2,576 (25.0%)
3rd Quartile	1,320 (23.5%)	416 (23.4%)	287 (28.3%)	553 (29.2%)	2,576 (25.0%)
2nd Quartile	1931 (34.4%)	169 (9.5%)	253 (25.0%)	223 (11.8%)	2,576 (25.0%)
1st Quartile (least deprived)	2038 (36.3%)	88 (4.9%)	283 (27.9%)	167 (8.8%)	2,576 (25.0%)
ICE_Race_ Quartile, *n* (%)					
1st Quartile (least racially privileged)	261 (4.6%)	1,255 (70.5%)	231 (22.8%)	829 (43.8%)	2,576 (25.0%)
2nd Quartile	1,081 (19.2%)	328 (18.4%)	436 (43.0%)	731 (38.6%)	2,576 (25.0%)
3rd Quartile	1886 (33.6%)	165 (9.3%)	262 (25.8%)	263 (13.9%)	2,576 (25.0%)
4th Quartile (most racially privileged)	2,389 (42.5%)	31 (1.7%)	85 (8.4%)	71 (3.7%)	2,576 (25.0%)
Maternal education, *n* (%)					
High school or less	592 (10.5%)	921 (51.8%)	162 (16.0%)	884 (46.7%)	2,559 (24.8%)
Some college; trade school	1,016 (18.1%)	525 (29.5%)	203 (20.0%)	585 (30.9%)	2,329 (22.6%)
Bachelor’s degree	2027 (36.1%)	223 (12.5%)	307 (30.3%)	260 (13.7%)	2,817 (27.3%)
Master’s degree or above	1982 (35.3%)	110 (6.2%)	342 (33.7%)	165 (8.7%)	2,599 (25.2%)

ICE_Race_, Index of Concentration at the Extremes for racial residential segregation.

**Table 3:** Maternal level of education, neighborhood deprivation index quartile, and index of concentrations of the extremes for race quartile in the ECHO cohort by maternal Census region of residence.

	Midwest	Northeast	South	West	Overall
	(*n* = 1639)	(*n* = 4242)	(*n* = 1540)	(*n* = 2883)	(*n* = 10304)
Neighborhood deprivation index, *n* (%)					
4th Quartile (most deprived)	411 (25.1%)	1,035 (24.4%)	528 (34.3%)	602 (20.9%)	2,576 (25.0%)
3rd Quartile	483 (29.5%)	926 (21.8%)	319 (20.7%)	848 (29.4%)	2,576 (25.0%)
2nd Quartile	409 (25.0%)	1,222 (28.8%)	239 (15.5%)	706 (24.5%)	2,576 (25.0%)
1st Quartile (least deprived)	336 (20.5%)	1,059 (25.0%)	454 (29.5%)	727 (25.2%)	2,576 (25.0%)
ICE_Race_ Quartile, *n* (%)					
1st Quartile (least racially privileged)	290 (17.7%)	839 (19.8%)	693 (45.0%)	754 (26.2%)	2,576 (25.0%)
2nd Quartile	260 (15.9%)	703 (16.6%)	405 (26.3%)	1,208 (41.9%)	2,576 (25.0%)
3rd Quartile	532 (32.5%)	862 (20.3%)	383 (24.9%)	799 (27.7%)	2,576 (25.0%)
4th Quartile (most racially privileged)	557 (34.0%)	1838 (43.3%)	59 (3.8%)	122 (4.2%)	2,576 (25.0%)
Maternal education, *n* (%)					
High school or less	436 (26.6%)	1,010 (23.8%)	621 (40.3%)	492 (17.1%)	2,559 (24.8%)
Some college; trade school	509 (31.1%)	886 (20.9%)	235 (15.3%)	699 (24.2%)	2,329 (22.6%)
Bachelor’s degree	351 (21.4%)	1,243 (29.3%)	358 (23.2%)	865 (30.0%)	2,817 (27.3%)
Master’s degree or above	343 (20.9%)	1,103 (26.0%)	326 (21.2%)	827 (28.7%)	2,599 (25.2%)

ICE_Race_, Index of Concentration at the Extremes for racial residential segregation.

For both linear and multinomial logistic regression modeling, we evaluated each SES measure of interest (level of maternal education, neighborhood deprivation, and racial segregation) using an unadjusted model (without adjustment for covariates), a co-adjusted model (adjusting the estimate for each SES exposure for the other SES exposures), and an adjusted model (adjusting for maternal age, marital/cohabitation status, parity, and child sex in addition to the other SES exposures).

Additionally, we conducted two types of sensitivity analyzes. In the first sensitivity analyzes, we explored the effect of further adjusting linear mixed effect models for maternal health conditions and behaviors previously associated with SES and gestational age at birth, including pre-pregnancy BMI and pregnancy-related conditions (preeclampsia, gestational hypertension, gestational diabetes) and prenatal substance use (tobacco, alcohol, marijuana), which could potentially confound or be on the causal pathway for any observed SES-gestational age at birth associations. In a second set of sensitivity analyzes, we performed ‘leave one out’ analysis in which we examined the point estimate for each SES measure of interest on completed gestational weeks in linear mixed effect modeling after excluding one cohort at a time.

We compared measures of association for unadjusted, co-adjusted, and further adjusted models to explore whether individual- and neighborhood-level measures of SES attenuate or potentiate observed associations by maternal health conditions and health behaviors. Based on *a priori* hypotheses that there would be substantial variation by maternal race and ethnicity and United States Census region, adjusted linear models were stratified by maternal race/ethnicity categories (Hispanic, non-Hispanic Black, non-Hispanic White, non-Hispanic Other Race) and by United States Census region (Northeast, Midwest, West, and South) to examine differences by strata.

Imputation was performed for missing data for the covariates of marital status, parity, pre-pregnancy BMI, prenatal substance use, gestational diabetes, and gestational hypertension, with multiple imputation (MI) by chained equations using the fully conditional specification with a discriminant function (27). Imputation models included gestational age; maternal education, race, ethnicity, and age; NDI; ICE_Race_; urbanicity; and Census region, with cohort membership as a classification variable. All statistical models were performed using non-imputed and imputed data. No substantial differences in estimated measures of association were observed when considering models based on non-imputed values (excluding cases with missing values) and multiple imputation values; thus, we chose to present models based on imputed data since parameter estimates were more stable. Estimates of association (Tables 4–7) combine estimates from five imputations.

**Table 4:** Linear regression modeling of maternal education, neighborhood deprivation and racial segregation on gestational age at birth (weeks) among singleton births 2000–2019 in the ECHO Cohort (*n* = 10,304 mother–child pairs).

Principle exposure	Unadjusted		Co-Adjusted		Adjusted	
	Estimate	95% CI	Estimate	95% CI	Estimate	95% CI
Maternal education						
High school or less	**−0.35**	**(−0.46,-0.24)**	**−0.27**	**(−0.39,-0.15)**	**−0.31**	**(−0.44,-0.18)**
Some college; AD; trade school	**−0.32**	**(−0.43,-0.21)**	**−0.27**	**(−0.39,-0.16)**	**−0.30**	**(−0.42,-0.18)**
Bachelor’s degree	−0.03	(−0.13,0.07)	−0.02	(−0.12,0.08)	−0.04	(−0.14,0.06)
Master’s degree or above	*Ref*	*Ref*	*Ref*	*Ref*	*Ref*	*Ref*
NDI Quartile						
4th Quartile (most deprived)	**−0.31**	**(−0.42,-0.2)**	−0.08	(−0.24,0.08)	−0.10	(−0.25,0.06)
3rd Quartile	−0.10	(−0.21,0)	0	(−0.12,0.11)	−0.01	(−0.13,0.1)
2nd Quartile	−0.03	(−0.13,0.08)	0.01	(−0.09,0.12)	0.01	(−0.1,0.11)
1st Quartile (least deprived)	*Ref*	*Ref*	*Ref*	*Ref*	*Ref*	*Ref*
ICE_Race_ Quartile						
1st Quartile (least racially privileged)	**−0.30**	**(−0.45,-0.16)**	−0.13	(−0.32,0.05)	−0.12	(−0.3,0.06)
2nd Quartile	−0.03	(−0.18,0.11)	0.03	(−0.12,0.18)	0.03	(−0.13,0.18)
3rd Quartile	0.01	(−0.12,0.14)	0.01	(−0.12,0.14)	0	(−0.13,0.14)
4th Quartile (most racially privileged)	*Ref*	*Ref*	*Ref*	*Ref*	*Ref*	*Ref*

^1^Model includes random intercept for ECHO cohort membership. No adjustment for covariates. ^2^Mutually adjusted for other principal exposure variables (level of education, neighborhood deprivation, racial segregation) and random intercept for ECHO membership. ^3^Further adjusted for maternal age, marital/cohabitation status, parity, and child sex. ICE_Race_, Index of Concentration at the Extremes for racial residential segregation. CI, confidence interval. Bold text indicates a statistically significant association.

**Table 5:** Multinomial regression modeling^1^ of maternal education, neighborhood deprivation and racial segregation on gestational age at birth categories (full term birth as referent category) among singleton births 2000–2019 in the ECHO cohort–secondary population excluding preterm only cohorts.

Principle exposure	Preterm			Early term		
	*N* = 746			*N* = 2,159		
	Unadjusted OR (95% CI)	Co-adjusted OR (95% CI)	Adjusted OR (95% CI)	Unadjusted OR (95% CI)	Co-adjusted OR (95% CI)	Adjusted OR (95% CI)
Maternal education						
High school or less	**1.62 (1.31,2.02)**	**1.34 (1.05,1.72)**	**1.59 (1.20,2.10)**	**1.36 (1.19,1.57)**	**1.19 (1.02,1.40)**	**1.26 (1.05,1.51)**
Some college; AD; trade school	**1.47 (1.18,1.83)**	**1.33 (1.05,1.68)**	**1.53 (1.19,1.97)**	1.09 (0.95,1.26)	1.02 (0.88,1.19)	1.06 (0.90,1.26)
Bachelor’s degree	0.92 (0.73,1.16)	0.91 (0.72,1.15)	0.97 (0.76,1.22)	1.00 (0.87,1.14)	0.99 (0.86,1.14)	1.01 (0.88,1.16)
Master’s degree or above	*Ref*	*Ref*	*Ref*	*Ref*	*Ref*	*Ref*
Neighborhood deprivation index						
4th Quartile (most deprived)	**1.79 (1.45,2.22)**	1.23 (0.90,1.69)	1.30 (0.94,1.79)	**1.43 (1.24,1.64)**	1.22 (0.99,1.50)	**1.24 (1.00,1.52)**
3rd Quartile	1.21 (0.97,1.52)	1.01 (0.79,1.31)	1.04 (0.81,1.35)	**1.17 (1.01,1.35)**	1.09 (0.93,1.28)	1.10 (0.93,1.29)
2nd Quartile	1.09 (0.86,1.37)	1.04 (0.82,1.32)	1.05 (0.83,1.33)	1.01 (0.88,1.17)	1.01 (0.87,1.17)	1.01 (0.87,1.17)
1st Quartile (least deprived)	*Ref*	*Ref*	*Ref*	*Ref*	*Ref*	*Ref*
ICE_Race_						
1st Quartile (least racially privileged)	**1.82 (1.46,2.26)**	**1.37 (1.02,1.84)**	1.29 (0.95,1.75)	**1.48 (1.29,1.71)**	1.20 (0.99,1.45)	1.18 (0.97,1.43)
2nd Quartile	**1.29 (1.02,1.62)**	1.18 (0.92,1.51)	1.13 (0.88,1.46)	**1.28 (1.11,1.48)**	**1.18 (1.01,1.38)**	1.16 (0.99,1.36)
3rd Quartile	1.18 (0.94,1.50)	1.19 (0.94,1.51)	1.19 (0.93,1.51)	1.20 (1.04,1.39)	**1.19 (1.03,1.38)**	**1.18 (1.02,1.37)**
4th Quartile (most racially privileged)	*Ref*	*Ref*	*Ref*	*Ref*	*Ref*	*Ref*

Principle Exposure	Late or post term		
	*N* = 1,245		
	Unadjusted OR (95% CI)	Co-adjusted OR (95% CI)	Adjusted OR (95% CI)
Maternal education			
High school or less	**0.78 (0.66,0.93)**	**0.78 (0.64,0.96)**	0.85 (0.68,1.08)
Some college; AD; trade school	**0.74 (0.62,0.89)**	**0.72 (0.59,0.87)**	**0.79 (0.64,0.96)**
Bachelor’s degree	0.97 (0.82,1.13)	0.93 (0.79,1.09)	0.95 (0.81,1.12)
Master’s degree or above	*Ref*	*Ref*	*Ref*
Neighborhood deprivation index			
4th Quartile (most deprived)	0.9 (0.75,1.08)	**1.59 (1.22,2.07)**	**1.64 (1.25,2.14)**
3rd Quartile	1.11 (0.94,1.32)	**1.41 (1.17,1.71)**	**1.40 (1.16,1.69)**
2nd Quartile	**1.21 (1.02,1.43)**	**1.26 (1.07,1.5)**	**1.25 (1.05,1.49)**
1st Quartile (least deprived)	*Ref*	*Ref*	*Ref*
ICE_Race_			
1st Quartile (least racially privileged)	**0.60 (0.50,0.71)**	**0.51 (0.40,0.65)**	**0.49 (0.38,0.63)**
2nd Quartile	**0.77 (0.65,0.91)**	**0.70 (0.59,0.84)**	**0.66 (0.55,0.79)**
3rd Quartile	**0.80 (0.68,0.94)**	**0.79 (0.67,0.94)**	**0.77 (0.65,0.91)**
4th Quartile (most racially privileged)	*Ref*	*Ref*	*Ref*

^1^Adjusted for or other principal exposure variables (level of education, neighborhood deprivation, racial segregation) and maternal age, marital/cohabitation status, parity, and child sex. ICE_Race_, Index of Concentration at the Extremes for racial residential segregation. CI, confidence interval. Bold text indicates a statistically significant association.

**Table 6:** Linear regression modeling of maternal education, neighborhood deprivation and racial segregation on gestational age at birth among singleton births 2000–2019 in the ECHO cohort–stratified by maternal race/ethnic category.

Principle exposure	Non-Hispanic white		Non-Hispanic black		Non-Hispanic other race		Hispanic	
	Estimate	95% CI	Estimate	95% CI	Estimate	95% CI	Estimate	95% CI
Maternal education								
High school or less	**−0.35**	**(−0.54,-0.16)**	0.17	(−0.34,0.68)	−0.08	(−0.53,0.37)	−0.37	(−0.76,0.01)
Some college; AD; trade school	**−0.32**	**(−0.47,-0.18)**	0.15	(−0.34,0.64)	−0.08	(−0.45,0.29)	**−0.45**	**(−0.82,-0.07)**
Bachelor’s degree	−0.04	(−0.15,0.07)	0.45	(−0.06,0.97)	−0.22	(−0.50,0.06)	−0.09	(−0.48,0.30)
Master’s degree or above	*Ref*	*Ref*	*Ref*	*Ref*	*Ref*	*Ref*	*Ref*	*Ref*
Neighborhood deprivation index								
4th Quartile (most deprived)	−0.07	(−0.32,0.17)	−0.43	(−1.03,0.16)	0.13	(−0.33,0.59)	−0.28	(−0.72,0.17)
3rd Quartile	0.03	(−0.10,0.16)	−0.31	(−0.88,0.26)	−0.07	(−0.41,0.27)	−0.24	(−0.64,0.16)
2nd Quartile	0.04	(−0.07,0.15)	−0.3	(−0.88,0.28)	0.07	(−0.25,0.39)	−0.29	(−0.70,0.12)
1st Quartile (least deprived)	*Ref*	*Ref*	*Ref*	*Ref*	*Ref*	*Ref*	*Ref*	*Ref*
ICE_Race_								
1st Quartile (least racially privileged)	0.08	(−0.21,0.36)	0.25	(−0.64,1.14)	−0.46	(−1.11,0.18)	0.06	(−0.67,0.79)
2nd Quartile	0.13	(−0.05,0.30)	0.24	(−0.63,1.11)	−0.1	(−0.66,0.47)	0.04	(−0.66,0.75)
3rd Quartile	0.03	(−0.10,0.17)	0.14	(−0.75,1.02)	−0.09	(−0.63,0.45)	0.06	(−0.63,0.75)
4th Quartile (most racially privileged)	*Ref*	*Ref*	*Ref*	*Ref*	*Ref*	*Ref*	*Ref*	*Ref*

^1^Model includes random intercept for ECHO cohort membership. No adjustment for covariates. ^2^Mutually adjusted for other principal exposure variables (level of education, neighborhood deprivation, racial segregation) and random intercept for ECHO membership. ^3^Further adjusted for maternal age, marital/cohabitation status, parity, and child sex. ICE_Race_, Index of Concentration at the Extremes for racial residential segregation. CI, confidence interval. Bold text indicates a statistically significant association.

**Table 7:** Linear regression modeling^1^ of maternal education, neighborhood deprivation and racial segregation on gestational age at birth among singleton births 2000–2019 in the ECHO cohort–stratified by Census Region of residence.

Principle exposure	NorthEast		West		Midwest		South	
	Estimate	95% CI	Estimate	95% CI	Estimate	95% CI	Estimate	95% CI
Maternal education								
High school or less	**−0.36**	**(−0.56,-0.15)**	−0.22	(−0.48,0.05)	**−0.39**	**(−0.74,-0.03)**	−0.32	(−0.69,0.05)
Some college; AD; trade school	**−0.32**	**(−0.51,-0.14)**	−0.21	(−0.42,0)	**−0.45**	**(−0.75,-0.15)**	−0.34	(−0.73,0.05)
Bachelor’s degree	−0.07	(−0.22,0.08)	0.02	(−0.16,0.20)	−0.21	(−0.48,0.07)	0.08	(−0.22,0.38)
Master’s degree or above	*Ref*	*Ref*	*Ref*	*Ref*	*Ref*	*Ref*	*Ref*	*Ref*
Neighborhood deprivation index								
4th Quartile (most deprived)	−0.11	(−0.37,0.16)	−0.04	(−0.33,0.26)	−0.16	(−0.56,0.23)	0.08	(−0.36,0.51)
3rd Quartile	−0.05	(−0.23,0.14)	0	(−0.22,0.21)	0.03	(−0.25,0.31)	0.17	(−0.2,0.55)
2nd Quartile	0.07	(−0.09,0.22)	−0.01	(−0.21,0.19)	−0.05	(−0.32,0.22)	−0.03	(−0.36,0.3)
1st Quartile (least deprived)	*Ref*	*Ref*	*Ref*	*Ref*	*Ref*	*Ref*	*Ref*	*Ref*
ICE_Race_								
1st Quartile (least racially privileged)	−0.03	(−0.34,0.27)	−0.26	(−0.74,0.23)	−0.04	(−0.43,0.34)	−0.25	(−0.84,0.34)
2nd Quartile	0.12	(−0.14,0.39)	−0.10	(−0.54,0.33)	−0.19	(−0.52,0.13)	0.09	(−0.46,0.63)
3rd Quartile	0.03	(−0.18,0.23)	−0.07	(−0.48,0.33)	−0.11	(−0.34,0.11)	0.13	(−0.41,0.66)
4th Quartile (most racially privileged)	*Ref*	*Ref*	*Ref*	*Ref*	*Ref*	*Ref*	*Ref*	*Ref*

^1^Adjusted for or other principal exposure variables (level of education, neighborhood deprivation, racial segregation) and maternal age, marital/cohabitation status, parity, and child sex and random intercept for ECHO cohort membership ICE_Race_, Index of Concentration at the Extremes for racial residential segregation. CI, confidence interval. Bold text indicates a statistically significant association.

All analyzes were performed using the R statistical software package, version 4.1.0 (R Foundation for Statistical Computing, Vienna, Austria). The *mice* package (61) was used for multiple imputation, *lme4* package was used linear mixed effects regression (60), and the *nnet* package (62) was used for multinomial logistic regression.

## 3 Results

### 3.1 Description of study population

The primary sample consisted of 10,304 ECHO participants from 34 ECHO cohorts who delivered a liveborn infant from 2000 through 2019 with available participants’ residential address during the prenatal period (Figure 2). The distribution of gestational age and the characteristics of the study population differed by maternal education level (Table 1). The mean gestational age at birth was 38.2 weeks (SD 3.4); approximately 54% of participants delivered full term, 13% preterm, 21% early term, and 12% late or post-term. Mean and median NDI values were higher (representing higher deprivation) while mean and median ICE_Race_ values were lower (representing a lower proportion of residents in the most racially privileged group, i.e., non-Hispanic White individuals) among mothers with lower educational levels (high school or less, some college) relative to those with higher educational levels (bachelor’s or master’s degree). Substantial proportions of the study population resided in the Northeast (41.2%) and West (28.0%) United States regions, with smaller proportions from the Midwest (15.9%) and South (14.9%). Overall, 18.4% of participants were Hispanic of any race; 54.5% were non-Hispanic White; 17.3% were non-Hispanic Black; and 9.9% were non-Hispanic Other Race. Participants’ mean age at birth was 30.5 years (SD 5.6) and approximately 35% had not given birth prior to the index delivery. Just over 40% of participants had a healthy BMI (18.5 to less than 25.0 kg/m^2^), with considerable variability by level of education (28.5% of those with a high school education or less had a healthy weight vs. 59.2% with a master’s degree or higher).

Approximately 5% of pregnancies in the study population were affected by preeclampsia or eclampsia, 4.5% by gestational hypertension, and 7.5% by gestational diabetes in the index pregnancy. Within the study population, maternal level of education, NDI, and ICE_Race_ varied by maternal race and ethnicity (Table 2) and United States Census region of residence (Table 3). Approximately half of non-Hispanic Black participants had a high school education or less compared with 10.5% of non-Hispanic White, 16.0% of non-Hispanic Other Race, and 46.7% of Hispanic participants. In addition, non-Hispanic Black participants were more likely to be in the first ICE_Race_ quartile and the fourth NDI quartile compared with all other race and ethnicity categories (Table 2). Those residing in the South had a greater frequency of a high school education or less and lower ICE_Race_ values, indicating a lower proportion of residents in the most racially privileged group (Table 3) compared with those residing in the Midwest, North, and West United States Census regions.

### 3.2 Gestational weeks at birth (continuous)–models for the overall primary sample

We found an association between the maternal education and gestational age at birth based on unadjusted, co-adjusted, and adjusted linear mixed effects regression models (Table 4).

Gestational age at birth was significantly lower among those with up to a high school diploma or less (−0.31 weeks, 95% CI: −0.44, −0.18) and some college (−0.30 weeks, 95% CI: −0.42, −0.18) relative to those with a master’s degree or higher across all models (unadjusted, co-adjusted, adjusted), with minimal change in the effect estimate after adjusting for the other principal exposure variables (NDI and ICE_Race_ quartile) and after including age, marital/cohabitation status, parity, and child sex. In the unadjusted model, a significantly lower gestational age at birth was observed among those with higher levels of neighborhood deprivation (third and fourth quartiles) relative to the first quartile (lowest deprivation), with attenuation of the effect with co-adjustment for the other SES exposure variables (maternal education and ICE_Race_ quartile) and virtually no change in the effect estimate with further adjustment for age, marital/cohabitation status, parity, and child sex. When considering the principal exposure of ICE_Race_ quartile, in the unadjusted model, a significantly lower gestational age at birth was found among those residing in census tracts in the first quartile (relatively lower proportions of non-Hispanic White individuals) relative to the fourth quartile (highest proportion of non-Hispanic White individuals; −0.30 weeks, 95% CI: −0.45, −0.16); with co-adjustment and in the adjusted model, no significant associations were observed between ICE_Race_ and gestational age at birth.

### 3.3 Gestational weeks at birth categories–secondary sample

**Figure 3:**
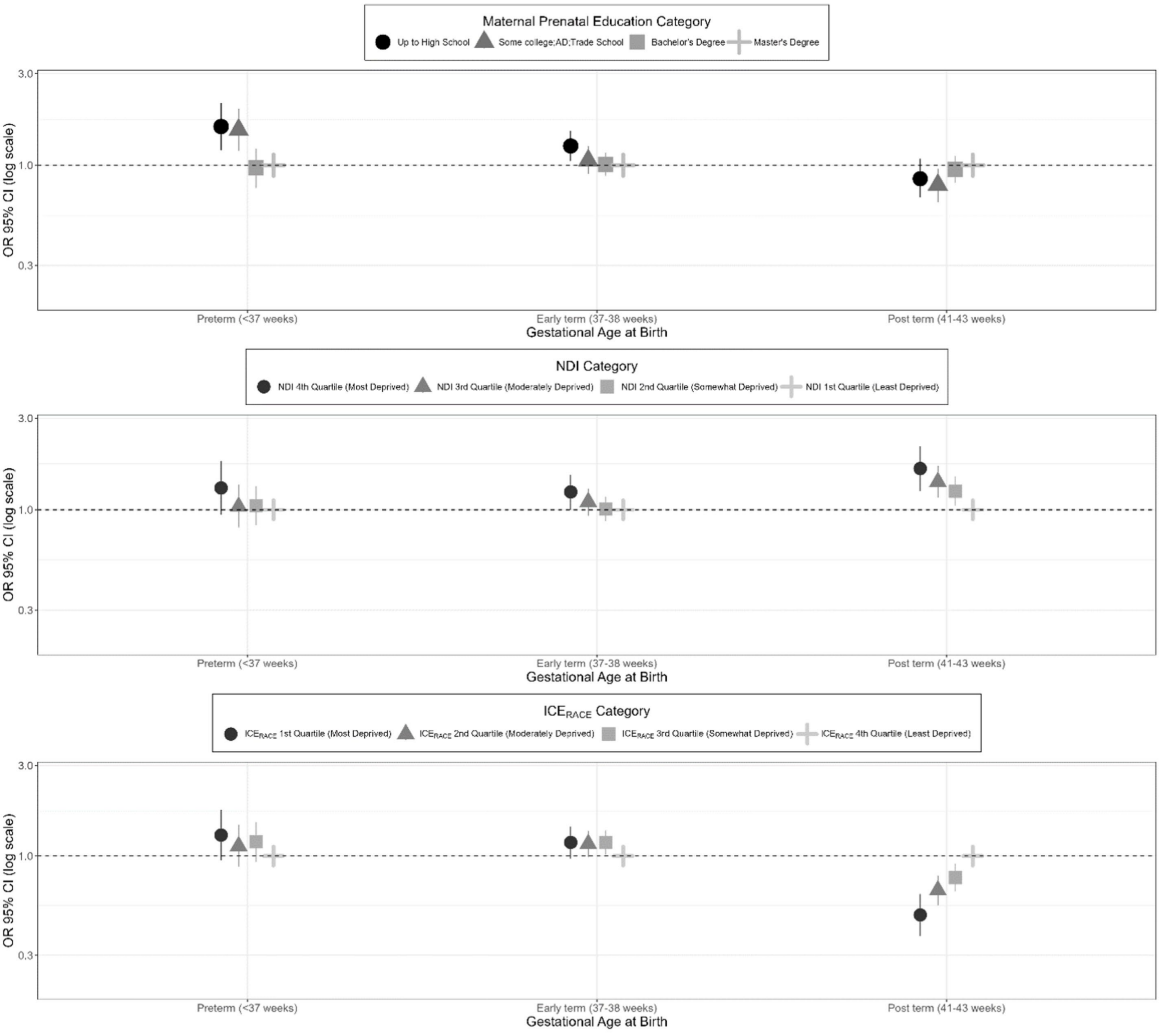
Adjusted odds ratios for gestational age at birth categories (full-term birth as referent category) among singleton births from 2000 to 2019 in the ECHO cohorts–secondary study population.

SES exposure variables (maternal education, neighborhood deprivation, and racial segregation) were associated with gestational age at birth outcomes (preterm, early-term, late- or post-term; full-term birth as referent) for the secondary sample in unadjusted, co-adjusted, and adjusted models (Table 5). These results are presented visually with adjusted ORs (aORs) and 95% CIs for the SES exposures and categories of gestational age at birth (Figure 3).

#### 3.3.1 Effect estimates for maternal education

In the unadjusted model utilizing the secondary study population, having a high school diploma or less vs. a master’s degree or above was associated with a significantly increased odds of preterm birth (OR 1.62, 95% CI: 1.31, 2.02) and early term birth (OR 1.36, 95% CI: 1.19, 1.57) and a decreased odds of late or post-term birth (OR 0.78, 95% CI: 0.66, 0.96). Similarly, in the unadjusted model, having some college was associated with a significantly increased odds of preterm birth (OR 1.47, 95% CI: 1.18, 1.83) and a significantly decreased odds of late- or post-term birth (OR 0.74, 95% CI: 0.62, 0.89). With co-adjustment for other principal exposure variables, effect estimates from the unadjusted model were somewhat attenuated. In the models that were additionally adjusted for maternal age, parity, marital/cohabitation status, and child sex, having a high school degree or less vs. a master’s degree or above remained associated with a significantly increased odds of preterm birth (aOR 1.59, 95% CI: 1.20, 2.10) and early term birth (aOR 1.26, 95% CI: 1.05, 1.51). Similarly, in the overall adjusted model, having some college was significantly associated with an increased odds of preterm birth (aOR 1.53, 95% CI: 1.19, 1.97). In the overall adjusted model, having some college vs. a master’s degree or above was also significantly associated with a decreased odds of late or post-term birth (aOR 0.79, 95% CI: 0.64, 0.96).

#### 3.3.2 Effect estimates for neighborhood deprivation index

For the secondary sample, an increased level of neighborhood deprivation (as indicated by the fourth NDI quartile) was associated with a significantly increased odds of preterm and early term birth in the unadjusted models. This association only remained significant after adjustment for the early term births (aOR 1.24, 95% CI: 1.00, 1.52). In addition, participants with increasing levels of neighborhood deprivation had a significantly increased odds of late or post term birth (OR 1.64, 95% CI 1.25, 2.15 for the fourth quartile compared to the first quartile) in the adjusted model.

#### 3.3.3 Effect estimates for neighborhood racial segregation

For the secondary sample, the unadjusted model showed a significant association between residence in a census tract in the first and second ICE_Race_ quartiles (least racially privileged) vs. the fourth quartile (most racially privileged) and preterm birth (aOR 1.82, 95% CI: 1.46, 2.26; aOR 1.29, 95% CI: 1.02, 1.62, respectively); however, these findings became non-significant after adjustment for covariates. The unadjusted model showed a significantly increased odds of early term birth among those in the first, second, and third ICE_Race_ quartiles, with some attenuation of the effect estimate in the co-adjusted and adjusted models, resulting in some non-significant results. In the unadjusted, co-adjusted, and adjusted models, there was consistently a significantly decreased odds of late or post-term birth among those residing in the first, second, and third ICE_Race_ quartiles compared to the fourth quartile (aOR 0.49, 95% CI 0.38, 0.64 for 1^st^ vs. 4^th^ quartile).

### 3.4 Gestational weeks at birth continuous–stratified by maternal race/ethnic category

In our primary study population, the association between SES exposure variables and gestational age at birth differed when stratified by maternal race/ethnic category (Table 6). We observed that gestational age at birth was lower for those with a high school education or less and some college vs. a master’s degree or above for only the non-Hispanic White category. No significant associations were observed between NDI and ICE_Race_ and gestational age at birth after stratification by maternal race/ethnicity.

### 3.5 Gestational weeks at birth continuous–models stratified by United States Census region

Associations between maternal education and gestational age at birth varied by United States Census region (Table 7). We observed that gestational age at birth was lower among those with a high school education or less and some college vs. a master’s degree or higher for those living in the NorthEast and Midwest during pregnancy. No significant associations were observed between NDI and ICE_Race_ and gestational age at birth after stratification by United States Census Region.

### 3.6 Gestational weeks at birth continuous–sensitivity analyzes

Estimates that incorporated adjustments for additional covariates related to maternal health conditions and substance use behaviors had a minimal impact on the results regarding the relationship between maternal education and neighborhood deprivation and gestational age at birth (Supplementary Table S1). Specifically, when considering the relationship between lower maternal education and gestational age at birth, additional adjustment for maternal pre-pregnancy BMI, pregnancy-related conditions (preeclampsia, gestational hypertension, gestational diabetes), and prenatal substance use did not meaningfully alter effect estimates. Associations between NDI and ICE_Race_ with gestational age at birth also did not differ after further adjustment.

In ‘leave one out’ analysis, the exclusion of one cohort at a time did not appreciably alter the point estimates for the associations between maternal education (Supplementary Figure S1A), NDI quartile (Supplementary Figure S1B), or ICE_Race_ (Supplementary Figure S1C) with completed gestational age at birth.

## 4 Discussion

This analysis of the combined effects of individual- and neighborhood-level measures of SES on gestational age at birth using data from 34 ECHO cohorts spanning 45 states and all United States Census regions suggests that lower maternal education was associated with an increased odds of a shorter pregnancy duration and preterm birth. The association between lower maternal education and shorter pregnancy duration was observed in the Northeast and Midwest Census regions only. When stratifying by maternal race and ethnicity, the significant associations between lower levels of maternal education and shorter pregnancy duration was seen for only non-Hispanic Whites. The lack of an association between lower maternal education and shorter pregnancy duration for racial and ethnic minority participants suggests that additional exposures (related to exposures to various forms of racism) may be more salient or may reflect that there were relatively few minority participants in the highest education category. Understanding the association between individual- and neighborhood-level factors with gestational age is critical for the identification and targeting of effective solutions to improve maternal and child health and health equity.

In our analysis of the combined effects of individual- and neighborhood-level measures of SES, neighborhood-level measures of SES were not associated with pregnancy duration overall. However, individuals living in more deprived neighborhoods (as operationalized by higher NDI quartile) had an increased odds of post term birth, and individuals living in higher racial privilege neighborhoods (conceptualized as a lower ICE_Race_ quartile reflecting a lower proportion of non-Hispanic White individuals in the census tract) had an decreased odds of post term birth.

Among our study participants, regional differences in racial segregation were evident in that 45.0% of those in the South Census region resided in the least racially privileged ICE_Race_ quartile compared with 26% or less in the other Census regions. When looking at the United States population overall, there remain significant regional differences in racial segregation levels, with higher levels in the United States South despite declines since 2000 (63).

Notably, in statistical models that additionally adjusted for maternal health conditions, including pre-pregnancy BMI and pregnancy-related conditions (preeclampsia, gestational hypertension, gestational diabetes) and substance use behaviors, little change occurred in the effect estimates for maternal education level and NDI quartile on gestational age at birth. This observation may suggest that these individual- and neighborhood-level measures of SES exert their influence partly through pathways that are not directly related to maternal health status and/or behaviors, which may include pathways mediated by psychosocial factors, social support and stress, environmental exposures, and access to healthy food and recreation (42, 64, 65). Additionally, consideration of prenatal substance use produced little change in the effect estimates for racial privilege of the census tract of residence. In contrast, adjustment for pre-pregnancy BMI and pregnancy-related conditions altered conclusions about the effect of ICE_Race_ quartile (racial segregation) on gestational age at birth; with the inclusion of these variables in the model, a lower ICE_Race_ quartile (i.e., lower proportion of non-Hispanic White individuals) associated with a decreased odds of preterm birth and attenuated the association with the increased odds of early-term birth. These findings suggest a complex relationship among pre-pregnancy BMI, pregnancy-related conditions, and racial segregation that warrants further investigation. For example, are neighborhoods with a lower proportion of non-Hispanic White individuals more communal or supportive in some way that influences gestational age at birth?

The relationship between a higher level of maternal education and a lower preterm birth risk has been previously documented in the United States (50, 51). Although our results were not significant for all racial/ethnic groups, point estimates suggested an association between increased levels of maternal education and decreased odds of preterm birth except among non-Hispanic Black participants. The lack of statistical power may have been due to smaller sample sizes for the race/ethnicity-specific strata. It is also possible, however, that the effect of maternal level of education is not as related to preterm birth among those of a race/ethnicity other than non-Hispanic White. One explanation for the persistence of racial/ethnic disparities after accounting for SES measures, such as maternal education, is that specified levels of SES are not equivalent across racial/ethnic groups (66, 67). At every level of education, Black women have lower earnings and less accumulated wealth than White women and face higher average costs for housing, food, and insurance while having more people dependent on their incomes; therefore, racial/ethnic groups may not be comparable at a given level of SES (68).

Previous studies employing neighborhood-level measures of deprivation largely support an association between greater deprivation and preterm birth. In one study, NDI was found to associate with preterm birth in four United States between 1995 and 2001 (28). Likewise, living in census tracts with high unemployment, low education, and high poverty has been found to associate with an increased odds of preterm birth among non-Hispanic White and non-Hispanic Black individuals, with larger effect sizes among non-Hispanic White individuals (26). A study from California of the Black-White disparity in preterm birth found that SES, measured by both maternal level of education and census tract poverty, contributed to disparate rates of preterm birth, especially for births less than 32 weeks’ gestation (27). Additional studies have found associations between preterm birth and census tract-level median household income in Louisiana (1997–1998) (33) and very high gentrification (percent change in education level, poverty level, and median household income) in New York City (2008–2010) (30). In contrast, one study found no increased risk of preterm birth with census tract-level median household income in Massachusetts (1996–2002) (30), and another study using a composite variable for neighborhood-level SES derived from seven census variables found no association between low neighborhood SES and preterm birth among a sample of 6,390 United States Black women after adjustment for individual-level characteristics (34).

Likewise, previous studies employing neighborhood-level measures of segregation largely support an association between segregation and preterm birth. A meta-analysis of 42 studies found that the association between segregation and preterm birth differed by race, with segregation (Black racial composition of the neighborhood) being associated with an increased odds of preterm birth primarily among non-Hispanic Black individuals (32). A systematic review of studies that considered segregation using the Index of Concentration at the Extremes for race and income combined (referred to as racialized economic segregation and typically represented as ICE_Race-Income_) on health outcomes found five studies examining the outcome of preterm birth; these studies support a significant association between higher racialized economic segregation and preterm birth (69). A meta-analysis of 42 studies found the association between segregation and preterm birth differed by race, with segregation being associated with an increased odds of preterm birth primarily among non-Hispanic Black women (32). Another study found significant variation in the effects of segregation on the rates of preterm birth according to the prevalence of preterm birth, noting that segregation heightens the risk for preterm birth in areas of low–preterm birth prevalence, but overall, has no effect in areas of high prevalence (70). The authors of this study conclude that segregation may be a secondary factor in areas of higher prevalence of preterm birth, which on average have fewer (need-adjusted) resources and a higher prevalence of other risk factors and conditions.

A growing body of research has assessed area-based measures of SES in conjunction with environmental exposures to investigate preterm birth from an environmental justice perspective. Such studies have found that exposures to air pollution and water pollution increase the risk for preterm birth, particularly among neighborhoods of low SES (71–73). Future studies should consider the effects of these potential explanatory factors on gestational age at birth for women according to race, ethnicity, and geography.

## 5 Strengths and limitations

This study addresses an important gap in the literature, namely the limited data on racial/ethnic and geographic variation in individual- and neighborhood-level measures of SES and gestational age at birth across the United States A strength of the ECHO-wide cohort is its heterogeneity with respect to race/ethnicity, geographic variation covering multiple states and regions, and SES. Another strength of the ECHO cohort data is the availability of maternal prenatal residential addresses, which allowed for geocoding and assignment of neighborhood-level measures of SES at the census tract. Publicly available birth record data does not allow for discernment of geography to the census tract level. Within the published literature, aside from our previously published disseminated meta-analysis (44), no other study has brought together data from multiple pediatric cohorts across multiple states to address whether individual- and neighborhood-level markers of SES associate with gestational age at birth across the United States Previous studies that have considered individual- and neighborhood-level markers of SES have mostly focused within a single state or have been conducted at a geographic level larger than census tract (such as city or county), which may not accurately characterize an individual’s true exposure to deprivation and segregation. Of note, however, use of the ECHO cohort data presents potential limitations of generalizability and/or selection bias in that the different cohorts have different inclusion/exclusion criteria and different distributions of participant race and ethnicity. Participants in the ECHO cohorts could differ from the general population, thereby impacting our ability to generalize findings.

An additional strength of the ECHO cohort data is the availability and wealth of data on maternal health conditions and pregnancy complications that are linked with gestational age at birth outcomes. This rich data available across the ECHO cohorts offer a unique opportunity to expand on the analyzes of geographic variation in gestational age outcomes based on natality files. Conversely, the heterogeneity of the ECHO cohorts presents limitations. There was considerable variability in the measurement methods of the different variables (e.g., maternal self-report vs. medical record), thus leading to the potential misclassification of outcomes (particularly given the variability in the range of methods that could be used to classify gestational age at births across participating cohorts) and exposures.

An important limitation of this study and many other studies is that we were unable to examine the duration of exposure to neighborhood deprivation and racial segregation, which would require the construction of longitudinal residential address data over time. Neighborhoods where a mother has lived over the life course, encompassing childhood, adolescence, and adulthood, may have significant effects on health later in life. Living in a poorer neighborhood as a youth may expose a mother to chronic stress that later increases the risk for preterm birth (74). A study of African American, White, and Latina births in California from 1982 to 2011 found that living in neighborhood poverty early in life and at adult time points was associated with mothers being at higher risk of preterm birth compared with living in a high-opportunity neighborhood throughout the life course (75). An additional limitation is that paternal level of education was not a variable that was widely available across the participating cohorts and was not included in this analysis. Paternal level of education has been independently associated with risk for preterm birth (76).

A further limitation of the present study is the lack of investigation into the effect of interpersonal racial discrimination on gestational age at birth outcomes in the United States A substantial body of research suggests that racial discrimination is an important stressor, especially for Black Americans, and that physiological responses to chronic stress related to interpersonal and systemic racial discrimination contribute to an excess risk of preterm birth (77–81). A systematic review of the literature, including 15 studies published between 2009 and 2015, observed that racial discrimination is a significant risk factor for delivering preterm (82). A multi-state analysis using a representative United States sample consisting of data from the Pregnancy Risk Assessment Monitoring System (PRAMS) from 2004 through 2012 observed that for non-Hispanic Black women, experiences of racism were significantly associated with a greater odds of delivering preterm (77). A study of African American women in Detroit, Michigan from 2009 to 2011 found that perceived racial microaggressions in the past year were associated with a higher risk of preterm birth (83). Another study of non-Latino White and Black births in California from 2011 to 2014 found that chronic worry about racial discrimination was associated with an increased risk of preterm birth; after adjusting for chronic worry about racial discrimination, the disparity in preterm birth among Black and White mothers was noticeably attenuated (84). This body of work highlights the need for future studies focused on the roles that these stressors play in these associations because education, SES, and other typically quantified prenatal exposures do not completely explain the racial disparities observed in preterm birth.

## 6 Conclusions and implications

The findings from this analysis indicate that both maternal education and neighborhood deprivation, representing individual- and neighborhood-level measures of SES, associate with preterm birth in the United States The findings suggest that interventions aimed at reducing preterm birth should include the promotion of higher educational attainment among women of reproductive age and multi-level community initiatives that foster community development. Additionally, the findings reinforce that for some racial and ethnic groups and geographic regions, factors other than higher educational attainment, such as racism, may be more salient exposures such that higher educational attainment and neighborhood privilege are not protective. Thus, interventions are needed that focus on achieving equitable access to the social determinants of health. In particular, there is a need for innovative social and economic policies and programs that support equal benefits from social resources for racial and ethnic minority individuals, including educational attainment. Such policies should focus on optimizing access to resources and assets and reducing societal and structural barriers that hinder racial and ethnic minority populations, including residential segregation and fewer and lower-quality educational opportunities (85). The Center for American Progress outlines a comprehensive framework for addressing United States racial and ethnic disparities in maternal and infant mortality through such approaches (18).

## Data availability statement

Select de-identified data from the ECHO Program are available through NICHD’s Data and Specimen Hub (DASH) (Eunice Kennedy Shriver National Institute of Child Health and Human Development, 2023). Information on study data not available on DASH, such as some Indigenous datasets, can be found on the ECHO study DASH webpage (Eunice Kennedy Shriver National Institute of Child Health and Human Development, 2023). Further enquires can be directed to the corresponding author’s.

## Ethics statement

The studies involving humans were conducted in accordance with the local legislation and institutional requirements. The ECHO study protocol was approved by the local and/or central ECHO Institutional Review Board. Written informed consent was provided by the participants’ legal guardians/next of kin (along with child assent as age-appropriate).

## Author contributions

AD, MB, LD, and MM contributed to conception and design of the study. MB and MM organized the database, performed the statistical analysis, and prepared tables and figures of results. AD and MM wrote the initial drafts of the manuscript. All authors contributed data for analysis and contributed to manuscript revision, read, and approved the submitted version of the manuscript.

## Group member of Environmental influences on Child Health Outcomes

ECHO Components—Coordinating Center: Benjamin DK, Smith PB, and Newby KL, Duke Clinical Research Institute, Durham, North Carolina; Data Analysis Center: Jacobson LP*, Johns Hopkins University Bloomberg School of Public Health, Baltimore, Maryland; Catellier D and Parker CB*, Research Triangle Institute, Durham, North Carolina; Person-Reported Outcomes Core: Gershon R and Cella D, Northwestern University, Evanston, Illinois.

ECHO Awardees and Cohorts—Alshawabkeh AN, Northeastern University, Boston, MA; Aschner J, Albert Einstein College of Medicine, Bronx, New York; Dabelea D. University of Colorado Denver, Denver, CO; Koinis Mitchell D, Deoni S, D’Sa V, Rhode Island Hospital, Providence RI; Duarte CS, New York State Psychiatric Institute, New York, NY; Dunlop AL, Emory University, Atlanta, GA; Elliott AJ, Avera Research Institute, Sioux Falls, SD; Ferrara A, Kaiser Permanente Northern California Division of Research, Oakland, CA; Breton C, University of Southern California, Los Angeles, CA; Hipwell A, University of Pittsburgh, Pittsburgh, PA; Karagas M, Geisel School of Medicine at Dartmouth, Lebanon, NH; Karr C, University of Washington, Department of Environmental and Occupational Health Sciences, Seattle, WA; Leve L, Prevention Science Institute, University of Oregon, Eugene, OR; Ganiban J, George Washington University, Washington, DC; Weiss ST, Brigham and Women’s Hospital, Boston, MA; McEvoy C, Oregon Health and Science University, Portland, OR; Lyall K, AJ Drexel Autism Institute, Philadelphia, PA; Oken E, Harvard Pilgrim Health Care Institute, Boston, MA; O’Shea M, University of North Carolina, Chapel Hill, NC; Kerver JM, Michigan State University, East Lansing, MI; Herbstman J, Columbia University Medical Center, New York, NY; Schantz S, University of Illinois, Beckman Institute, Urbana, IL; Stanford J, University of Utah, Salt Lake City, UT; Wright RJ, Icahn School of Medicine at Mount Sinai, New York, NY; Huddleston K, George Mason University, Fairfax, VA; Sathyanarayana S, Seattle Children’s Research Institute, Seattle, WA; Gern J, University of Wisconsin, Madison WI; Merhar S, Cincinnati Children’s Hospital Medical Center, Cincinnati, OH; Ren C, Indiana University, Riley Hospital for Children, Indianapolis, IN; Reynolds A, University of Buffalo, Jacobson School of Medicine and Biomedical Sciences, Buffalo, NY; Keller R, University of California, San Francisco; Pryhuber G, University of Rochester Medical Center, Rochester, NY; Duncan A, University of Texas Health Sciences Center, Houston, TX; Moore P, Vanderbilt Children’s Hospital, Nashville, TN; Lampland A, Children’s Hospital and Clinic Minneapolis, MN; Wadhawan R, Florida Hospital for Children, Orlando, FL; Wagner C, Medical University of South Carolina, Charleston, SC; Keller R, University of Arkansas for Medical Science; Hudak M, University of Florida College of Medicine, Jacksonville, FL; Mayock D, University of Washington, Seattle, WA; Washburn L, Wake Forest University School of Medicine, Winston Salem, NC; Canino G, University of Puerto Rico, San Juan, PR; Croen L, Kaiser Permanente Northern California Division of Research, Oakland, CA; Detroit, MI and Zoratti E, Henry Ford Health System; Seroogy C and Bendixsen C, Marshfield Clinic Research Institute, Marshfield, WI; Johnson C, Henry Ford Health System, Detroit, MI; Bastain T, Farzan S, and Habre R, University of Southern California, Los Angeles, CA; Mason A, University of Tennessee Health Science Center, Memphis, TN; Women and Infants Hospital of Rhode Island, Providence RI, Lester B; Carter B, Children’s Mercy, Kansas City, MO; Pastyrnak S, Helen DeVos Children’s Hospital, Grand Rapids, MI; Neal C, Kapiolani Medical Center for Women and Children, Providence, RI; Smith L, Los Angeles Biomedical Research Institute at Harbor-UCLA Medical Center, Los Angeles CA; Helderman J, Wake Forest University School of Medicine, Winston Salem, NC; O’Connor G, Boston University Medical Center, Boston, MA; Zeiger R, Kaiser Permanente, Southern California, San Diego, CA; Bacharier L, Washington University of St. Louis, St Louis, MO; Volk H, Johns Hopkins Bloomberg School of Public Health; O’Connor T, University of Rochester Medical Center Rochester, NY; Simhan H, University of Pittsburgh Medical Center, Magee Women’s Hospital, Pittsburgh, PA; Vaidya R, Baystate Children’s Hospital, Springfield, MA; Obeid R, Beaumont Health Medical Center, Royal Oak, MI; Rollins C, Boston Children’s Hospital, Boston, MA; Bear K, East Carolina University Brody School of Medicine, Greenville, NC; Pastyrnak S, Helen DeVos Children’s Hospital, Grand Rapids, MI; Lenski, M, Michigan State University College of Human Medicine, East Lansing, MI; Msall M, University of Chicago, Chicago IL; Frazier J, University of Massachusetts Medical School, Worcester, MA; Washburn, L, Wake Forest Baptist Health (Atrium Health), Winston Salem, NC; Montgomery A, Yale School of Medicine, New Haven, CT; Barone, C, Henry Ford Health System, Detroit, MI; McKane, P, Michigan Department of Health and Human Services, Lansing, MI; Paneth N, Michigan State University, East Lansing, MI; Elliott, M, University of Michigan, Ann Arbor, MI; Woodruff T, University of California, San Francisco, San Francisco, CA; Porucznik C, University of Utah, Salt Lake City, UT; Silver R, University of Utah, Salt Lake City, UT; Trasande L, New York School of Medicine, New York, NY; Bosquet-Enlow M, Boston Children’s Hospital, Boston MA; Bush N, University of California, San Francisco, San Francisco CA; Nguyen R, University of Minnesota, Minneapolis, MN; Rochester, NY and Barrett E, University of Rochester Medical Center; Miller R, Columbia University Medical Center, New York, NY.
