# Supplementary material for: Association of maternal education, neighborhood deprivation, and racial segregation with gestational age at birth by maternal race/ethnicity and United States Census region in the ECHO cohorts
Source: Front Public Health. 2023 Nov 30;11:1165089. doi: 10.3389/fpubh.2023.1165089 (PMC10719953; doi:10.3389/fpubh.2023.1165089)
Supplement: Supplementary file 1 [file Data_Sheet_1.docx]

**Supplementary Table 1.** Linear Regression Modeling^1,2,3^ of Maternal Education, Neighborhood Deprivation and Racial Segregation on Gestational Age at Birth Among

Singleton Births 2000-2019 in the ECHO Cohort

|  | **Adjusted^1^** | | **Adjusted^2^** | | **Adjusted^3^** | |
| --- | --- | --- | --- | --- | --- | --- |
|  | **Estimate** | **95% CI** | **Estimate** | **95% CI** | **Estimate** | **95% CI** |
| ***Maternal Education*** |  |  |  |  |  |  |
| High School or Less | **-0.31** | **(-0.44,-0.18)** | **-0.27** | **(-0.4,-0.13)** | **-0.27** | **(-0.41,-0.14)** |
| Some College; AD; Trade School | **-0.30** | **(-0.42,-0.18)** | **-0.27** | **(-0.39,-0.15)** | **-0.27** | **(-0.39,-0.15)** |
| Bachelor's Degree | -0.04 | (-0.14,0.06) | -0.02 | (-0.12,0.08) | -0.02 | (-0.12,0.08) |
| Master's Degree or Above | *Ref* | *Ref* | *Ref* | *Ref* | *Ref* | *Ref* |
| ***NDI Quartile*** |  |  |  |  |  |  |
| 4th Quartile (most deprived) | -0.10 | (-0.25,0.06) | -0.08 | (-0.24,0.07) | -0.08 | (-0.24,0.07) |
| 3rd Quartile | -0.01 | (-0.13,0.10) | -0.01 | (-0.12,0.11) | -0.01 | (-0.13,0.11) |
| 2nd Quartile | 0.01 | (-0.01,0.11) | 0.02 | (-0.09,0.12) | 0.02 | (-0.09,0.12) |
| 1st Quartile (least deprived) | *Ref* | *Ref* | *Ref* | *Ref* | *Ref* | *Ref* |
| ***ICE_Race_ Quartile*** |  |  |  |  |  |  |
| 1st Quartile (least racially privileged) | -0.12 | (-0.30,0.06) | -0.07 | (-0.25,0.12) | -0.06 | (-0.25,0.12) |
| 2nd Quartile | 0.03 | (-0.13,0.18) | 0.05 | (-0.10,0.21) | 0.06 | (-0.10,0.21) |
| 3rd Quartile | 0 | (-0.13,0.14) | 0.01 | (-0.12,0.14) | 0.01 | (-0.12,0.14) |
| 4th Quartile (most racially privileged) | *Ref* | *Ref* | *Ref* | *Ref* | *Ref* | *Ref* |

^1^ Adjusted for principal exposure variables (education, neighborhood deprivation, racial segregation) and for maternal age, marital/cohabitation status, parity, and child sex and random intercept for ECHO cohort membership

^2^ Further adjusted for maternal pre-pregnancy BMI and pregnancy-related medical conditions (preeclampsia, gestational hypertension, gestational diabetes)

^3^ Further adjusted for maternal prenatal substance use (alcohol, tobacco, marijuana)

ICE_Race_, Index of Concentration at the Extremes for racial residential segregation. CI, confidence interval

**Supplementary Figure 1A.** Linear Regression Modeling^1^ of Maternal Education on Gestational Age at Birth (Weeks) Among Singleton Births 2000-2019 in the ECHO Cohort in “Leave One Out” Analysis (in which one cohort of the 34 is left out in each iteration)


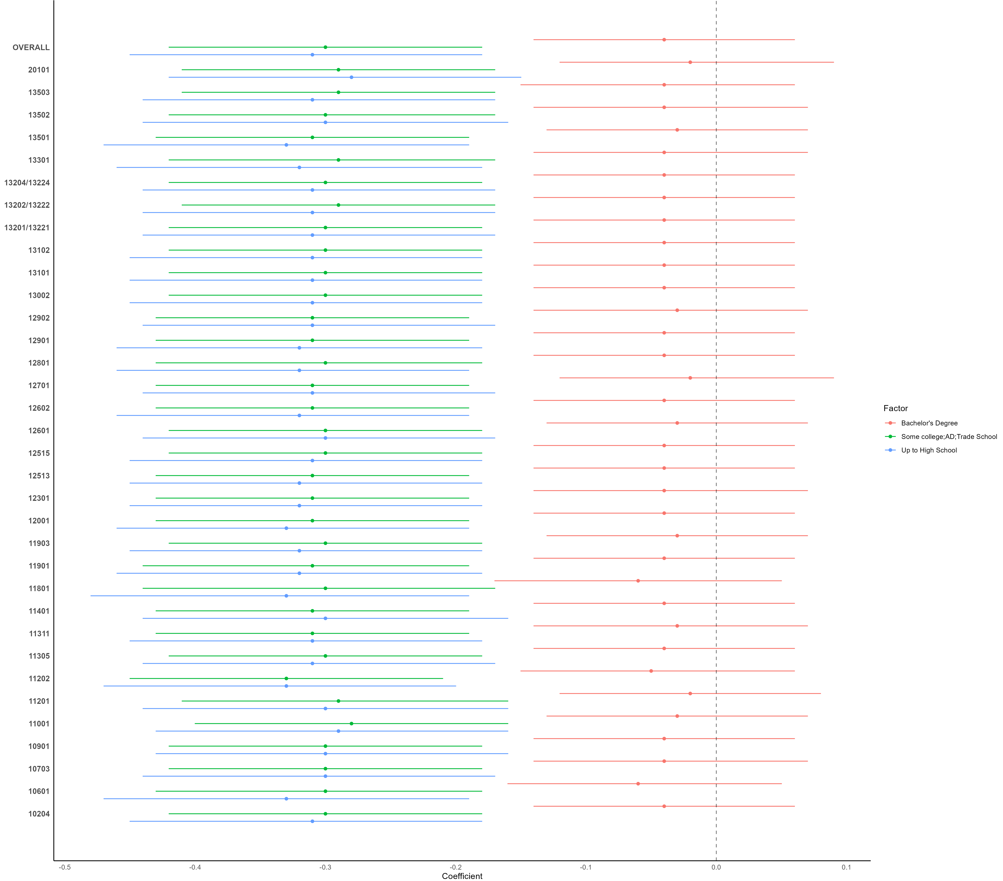


^1^ Adjusted for principal exposure variables (education, neighborhood deprivation, racial segregation) and for maternal age, marital/cohabitation status, parity, and child sex and random intercept for ECHO cohort membership

**Supplementary Figure 1B.** Linear Regression Modeling^1^ of Neighborhood Deprivation on Gestational Age at Birth (Weeks) Among Singleton Births 2000-2019 in the ECHO Cohort in “Leave One Out” Analysis (in which one cohort of the 34 is left out in each iteration)


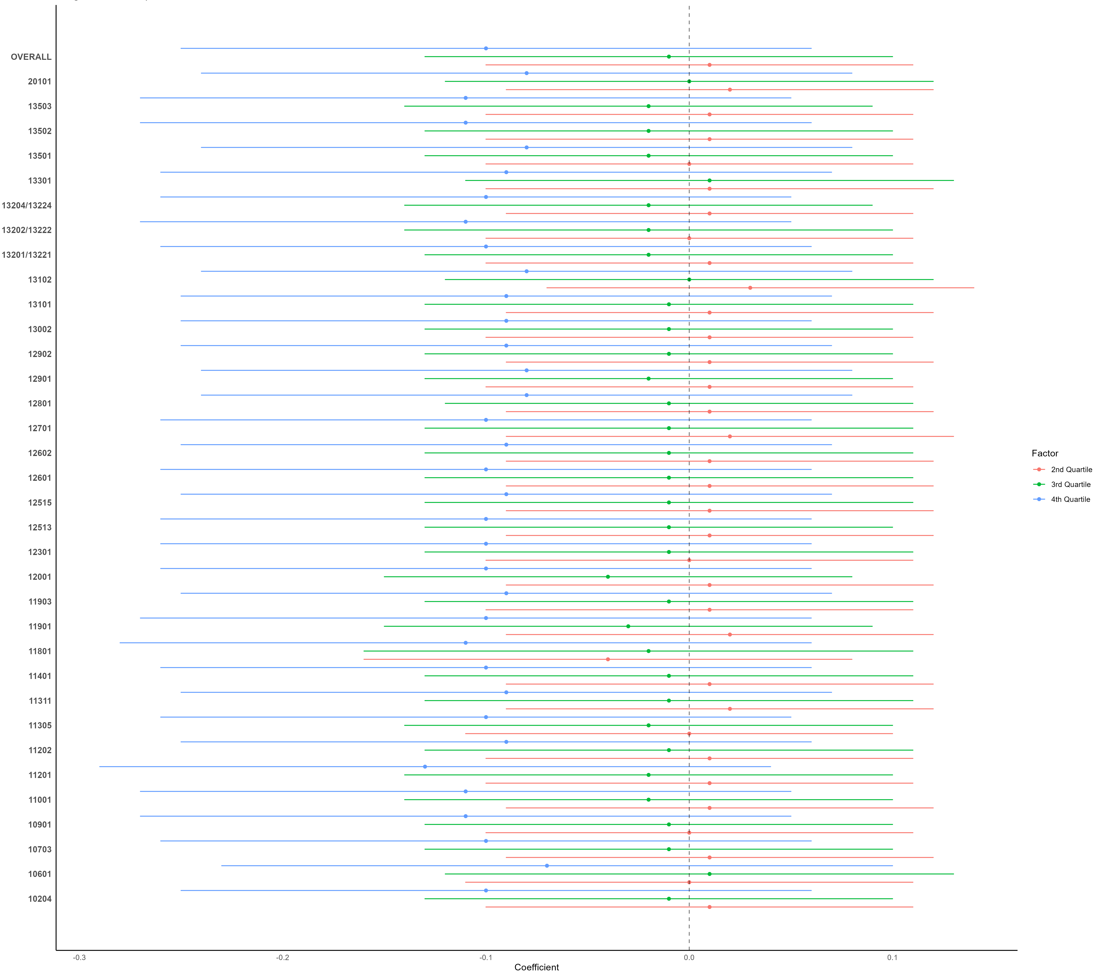


^1^ Adjusted for principal exposure variables (education, neighborhood deprivation, racial segregation) and for maternal age, marital/cohabitation status, parity, and child sex and random intercept for ECHO cohort membership

**Supplementary Figure 1C.** Linear Regression Modeling^1^ of Racial Segregation on Gestational Age at Birth (Weeks) Among Singleton Births 2000-2019 in the ECHO Cohort in “Leave One Out” Analysis (in which one cohort of the 34 is left out in each iteration)


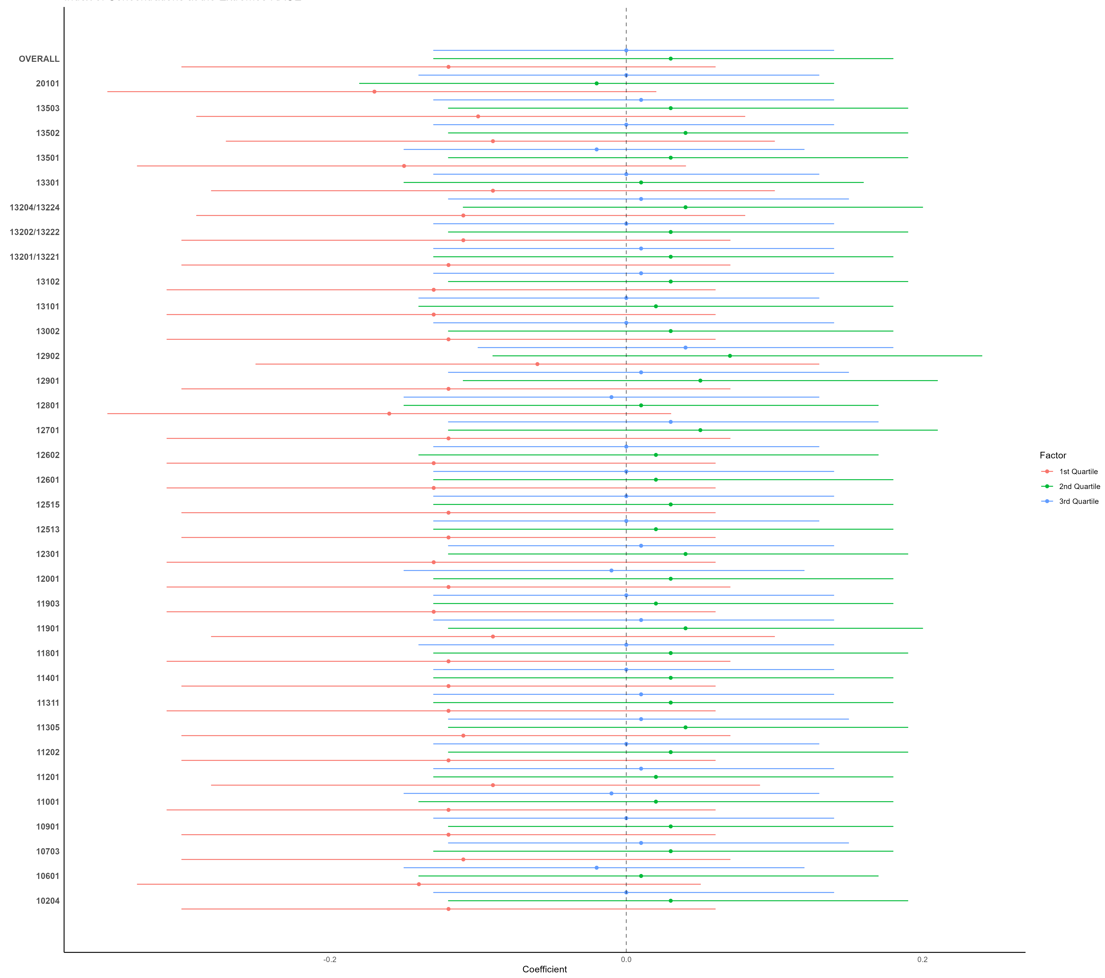


^1^ Adjusted for principal exposure variables (education, neighborhood deprivation, racial segregation) and for maternal age, marital/cohabitation status, parity, and child sex and random intercept for ECHO cohort membership
